# GLiMMPS: robust statistical model for regulatory variation of alternative splicing using RNA-seq data

**DOI:** 10.1186/gb-2013-14-7-r74

**Published:** 2013-07-22

**Authors:** Keyan Zhao, Zhi-xiang Lu, Juw Won Park, Qing Zhou, Yi Xing

**Affiliations:** 1 Department of Microbiology, Immunology, and Molecular Genetics, University of California, Los Angeles, CHS 33-228, 650 Charles E. Young Drive South, Los Angeles, CA 90095, USA; 2 Department of Internal Medicine, University of Iowa, 200 Hawkins Drive, Iowa City, IA 52242, USA; 3 Department of Statistics, University of California, Los Angeles, 8125 Math Sciences Building, Los Angeles, CA 90095, USA

**Keywords:** RNA-seq, alternative splicing, sQTL, exon, generalized linear mixed model

## Abstract

To characterize the genetic variation of alternative splicing, we develop GLiMMPS, a robust statistical method for detecting splicing quantitative trait loci (sQTLs) from RNA-seq data. GLiMMPS takes into account the individual variation in sequencing coverage and the noise prevalent in RNA-seq data. Analyses of simulated and real RNA-seq datasets demonstrate that GLiMMPS outperforms competing statistical models. Quantitative RT-PCR tests of 26 randomly selected GLiMMPS sQTLs yielded a validation rate of 100%. As population-scale RNA-seq studies become increasingly affordable and popular, GLiMMPS provides a useful tool for elucidating the genetic variation of alternative splicing in humans and model organisms.

## Background

Alternative splicing (AS) is the process by which exons from precursor mRNA transcripts are differentially included during splicing, resulting in different mature mRNA isoforms from a single gene locus [1]. AS is a major contributor to the control of gene expression and protein diversity. More than 90% of human genes are alternatively spliced [2]. Changes in the relative ratio of alternatively spliced isoforms of a single gene can have significant phenotypic consequences and cause various diseases [3,4].

The control of AS is mediated through extensive protein-RNA interactions involving *cis *regulatory elements and *trans *acting factors [5]. Genetic polymorphisms that alter *cis *splicing regulatory elements can result in difference of alternative splicing among human individuals and subsequently affect gene expression or protein activity. Increasing evidence suggests that such natural variation of alternative splicing can influence complex traits or modify disease risks [6]. For example, genetic variation of alternative splicing in the sodium channel gene *SCN1A *can influence the response to antiepileptic drugs [7]. To date, most genome-wide surveys of alternative splicing variation in human populations were carried out on the HapMap lymphoblastoid B cell lines (LCLs), whose genomic variants have been extensively characterized by the HapMap [8] and 1000 Genomes projects [9]. The first few studies utilized the Affymetrix exon array with approximately 6 million exon-targeted probes [10-12]. In these studies, the microarray probe intensities of individual exons were compared to those of whole genes to quantify exon inclusion levels and then associations with single-nucleotide polymorphisms (SNPs) were tested to identify splicing Quantitative Trait Loci (sQTLs). Another study used the same exon array platform to characterize tissue-specific control of alternative splicing in brain and peripheral blood mononuclear cell samples [13]. These studies have shed light on the prevalence and functional importance of alternative splicing variation in human populations. The development of the high-throughput RNA sequencing (RNA-seq) technology has provided a powerful alternative to splicing sensitive microarray for exon level expression quantification. RNA-seq has several advantages compared to microarray, including a greater dynamic range of exon expression levels, the ability to detect novel transcripts not probed on the array, the ability to better quantify exon inclusion levels, single nucleotide level resolution, and less confounding effects from polymorphisms on the target exons [14,15]. Several studies have used the RNA-seq technology to characterize transcriptome variation in HapMap LCLs at the whole-gene and/or individual exon level. Pickrell et al. and Montgomery et al. used low-coverage (4-25 million short reads per individual) single-end and paired-end RNA-seq to characterize gene expression and splicing in LCLs derived from 69 Nigerian [16] and 60 CEU (Utah residents of European descent from CEPH-Centre d'Etude du Polymporphisme Humain) [17] individuals. Cheung et al. independently generated an RNA-seq dataset on 41 CEU individuals at a deeper coverage of 28.4-66 million single-end reads per individual, although the authors restricted their data analysis to expression QTLs [18].

**Figure 1:**
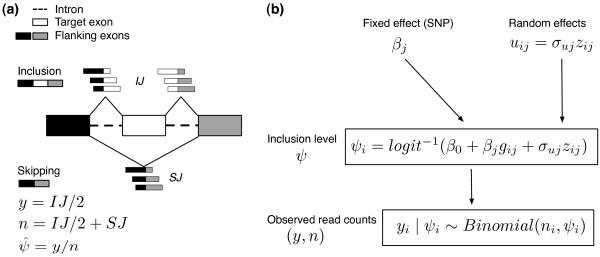
**Schematic outline of GLiMMPS**. (**a**) RNA-seq reads mapped to splice junctions of alternatively spliced exons are used for estimating exon inclusion levels *ψ*. Shown here is a schematic illustration using the skipped exon (SE) type of alternative splicing events as the example. White, sQTL target exon; black and gray, flanking exons. The inclusion junction (IJ) reads consist of reads mapped to the upstream and downstream splice junctions of the exon inclusion isoform, while the skipping junction (SJ) reads are reads mapped to the skipping splice junction of the exon skipping isoform. **(b) **Illustration of the GLiMMPS statistical model. SNP genotype effect is modeled as fixed effect *β_j_*. The overdispersion is modeled as individual level random effect $u_{ij}$.

Despite the novel findings in these pioneering RNA-seq studies, the statistical models applied for sQTL detection were simple linear regression models (lm) and did not model all the relevant information contained in the complex RNA-seq data. Montgomery et al. used the exon read counts as the phenotype and carried out spearman correlation analysis with the genotypes [17], while Pickrell et al. used the percentage of the exon read counts over total gene read counts as the quantitative trait and carried out linear regression over genotypes [16]. Neither approach directly estimated the percent inclusion levels of target exons. Moreover, by treating the exon expression measurement as a point estimate, neither approach considered the variability of RNA-seq read count that strongly affects the uncertainties in estimates of exon splicing activities [14]. Here we report a novel method GLiMMPS (Generalized Linear Mixed Model Prediction of sQTL) for robust detection of sQTLs from RNA-seq data. The GLiMMPS model takes into account the individual variation of exon-specific read coverage as well as the prevalent overdispersion of simple statistical models when applied to RNA-seq data [19,20]. Importantly, GLiMMPS uses the reads information from both exon inclusion and skipping isoforms to model the estimation uncertainty of exon inclusion level, instead of treating the exon inclusion level as a point estimate in sQTL analysis (see Materials and methods and Figure 1 for details). Using both simulated and real RNA-seq datasets, we demonstrate that GLiMMPS outperforms competing statistical models (linear model and generalized linear model), and identifies sQTLs at a low false positive rate as indicated by extensive RT-PCR tests.

## Results

### AS in the human population measured from RNA-seq data

We obtained the RNA-seq data from two published studies on the CEU population (of European ancestry) by Cheung et al. [18] and Montgomery et al. [17]. Cheung et al. generated 28.4-66 million 50 bp single-end reads per individual on 41 CEU samples, while Montgomery et al. generated 3.5-17.1 million 37 bp paired-end reads per individual on 60 CEU samples. Twenty-nine individuals were shared between the two datasets. Because of the higher sequencing depth in the Cheung et al. dataset, the analysis in this manuscript was primarily conducted on the Cheung et al. data (referred to hereafter as the CEU dataset). We also used the low-coverage Montgomery et al. data (referred to hereafter as the CEU2 dataset) to evaluate the concordance of results between the two CEU sample datasets.

The RNA-seq reads were mapped to the human genome (hg19) and transcriptome (Ensembl gene annotation r65) using the software Tophat [21]. To estimate the exon inclusion level (denoted as *ψ *for PSI, that is Percent Spliced In) from RNA-seq data, we used sequence reads mapped to splice junctions compiled from both the splice junctions in Ensembl gene annotations as well as the novel junctions found by Tophat. Based on the AS patterns, we classified the AS events into four categories (Figure S1 in Additional file 1): skipped exon (SE), alternative 5' splice site (A5SS), alternative 3' splice site (A3SS), and mutually exclusive exons (MXE). Using all splice junction reads, we can obtain a point estimate of the exon inclusion level ($\hat{\psi }$). We illustrate the estimate of *ψ *in our model using the SE event as the example (Figure 1a). Suppose *IJ *and *SJ *represent read counts of inclusion and skipping splice junctions, respectively, because *IJ *can come from both the upstream junction and the downstream junction, we treat the effective read count from the exon inclusion isoform y=IJ/2 and the effective read count from the exon skipping isoform as *SJ*. Given an observed total junction read count of *n= IJ/2+SJ*, the point estimate of $\hat{\psi }=y/n$. The median and coefficient of variation (CV) of $\hat{\psi }$ of skipped exons from CEU and CEU2 (with |Δψ|≥0.1 within each of the two populations, see Materials and methods) are highly correlated with a Pearson correlation coefficient of 0.99 and 0.90, respectively, suggesting that the point estimate of $\hat{\psi }$ provides a reasonable approximation to the exon inclusion level. However, we also noted that the total counts of splice junction reads for the same alternatively spliced exon typically vary substantially across different individuals (Figure S2 in Additional file 1), possibly due to the intrinsic randomness of RNA-seq technology as well as individual variation in gene expression levels. Such variability of read depth is expected to differentially affect the reliability of $\hat{\psi }$ estimates across individuals. This motivated us to develop an improved statistical model that explicitly considers the variation of RNA-seq read depth across individuals.

### Statistical model and simulation study of GLiMMPS

We first attempted to handle the individual variation of RNA-seq read depth by extending the previously used linear model (lm) [16] to a generalized linear model (glm) with a logit link function, which assumes the read count from the exon inclusion isoform (y) follows a binomial distribution y|ψ~Binomial(n,ψ), and logit(*ψ*) is linearly modeled by the SNP effect. This simple logistic regression model assumes that *ψ *is correctly modeled and thus: $E\left(y_{i}\right)=n_{i}\psi_{i}$, and $Var\left(y_{i}\right)=n_{i}\psi_{i}\left(1-\psi_{i}\right)$. However, we found that overdispersion (inflation of variance) is widespread in the experimental data (Supplementary Methods in Additional file 1). For the top sQTLs (Type I error <1% based on permutation) identified from glm in the CEU dataset, >90% sQTLs have significant overdispersion (Figure S3 in Additional file 1).

To model the overdispersion, we developed GLiMMPS, a generalized linear mixed model for detecting sQTLs. To deal with the overdispersion in the generalized linear model, we model the extra variance of *ψ *as a random effect for each individual *i *in the regression model with random effects, $u_{ij}\sim N\left(0,\sigma_{uj}^{2}\right)$[22]. Let $u_{ij}=\sigma_{uj}^{}z_{ij}$, where $z_{ij}\sim N\left(0,1\right)$, *β_j _*denoting the fixed effect for SNP *j*, the second level of the model can be written as: $\psi_{i}=\text{log}it^{-1}\left(\beta_{0}+\beta_{j}g_{ij}+\sigma_{uj}^{}z_{ij}\right)$. GLiMMPS is essentially a hierarchical model that considers both the read depth variation and the exon inclusion level variation within the same genotype groups (Figure 1b). Details of the lm, glm, and GLiMMPS models were described in Materials and methods and Supplementary Methods in Additional file 1.

**Figure 2:**
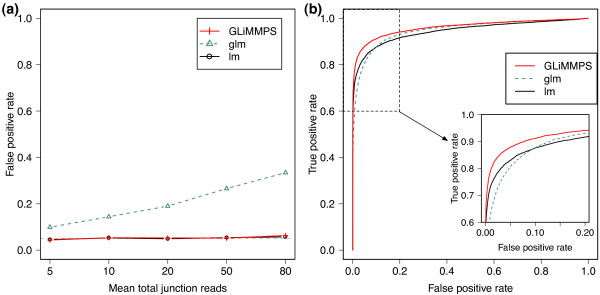
**Performance evaluation of different statistical models using simulated data**. **(a) **The observed false positive rate at the significance level of 0.05 for the linear model (lm), generalized linear model (glm), and GLiMMPS. Data were simulated with different sequencing depth with mean total junction reads ranging from 5 to 80, as described in Supplementary Methods in Additional file 1. **(b) **Receiver operating characteristic (ROC) curve analysis demonstrates that GLiMMPS outperforms the lm and glm models. The ROC curve plots the fraction of true positives called correctly and the fraction of false positives called incorrectly using P-values from each model. The zoomed-in figure shows the part of the ROC curve where the false positive rate is in the range of (0, 0.2).

We first conducted simulation studies to compare the power and robustness of GLiMMPS to lm and glm. We simulated splice junction read counts with various levels of read depth, difference of exon inclusion levels among genotype groups, and overdispersion mimicking the parameter distributions in the CEU dataset (Figure S4 in Additional file 1). We simulated 10,000 data points for each read depth with mean total splice junction reads ranging from 5 to 80. Data were simulated with 20% data points having genotype effects as distributed from the CEU dataset and the remaining 80% having no difference in exon inclusion levels among genotypes (see details in Supplementary Methods in Additional file 1). Note that the simulation data generated through this procedure are not inherently biased towards any of the statistical models tested. Using the 80% simulated data points with no SNP effect under various read depth, we evaluated the false positive rates (type I errors) at 5% significance level. The false positive rates of GLiMMPS and lm are always close to the nominal significance level, while glm has a highly inflated false positive rate, especially for data with large total splice junction reads (Figure 2a). This confirms that it is essential to incorporate overdispersion in the hierarchical model to avoid the inflation of *P *values. We also computed the receiver operating characteristic (ROC) curves by combining all the simulated data with or without SNP effects. The ROC curves show that GLiMMPS outperforms the lm and glm models (Figure 2b), especially in the most critical part of the ROC curve where the false positive rate is low. The true positive rate of GLiMMPS is approximately 5% to 20% higher than those of lm and glm when the false positive rate ranges from 0.01 to 0.1 (Figure 2b, inset). Furthermore, to model the non-uniformity and bias in sequence-specific sequencing preferences in RNA-seq data, we performed an additional simulation analysis. Specifically, for each exon inclusion or skipping splice junction we rescaled the original simulated count by a random scaling factor ranging from 0.5 to 2, with 10% variation in the scaling factor for the same splice junction across different individuals. We observed no change in the performance of GLiMMPS as compared to lm and glm (data not shown).

### Performance of GLiMMPS in real human RNA-seq data

**Figure 3:**
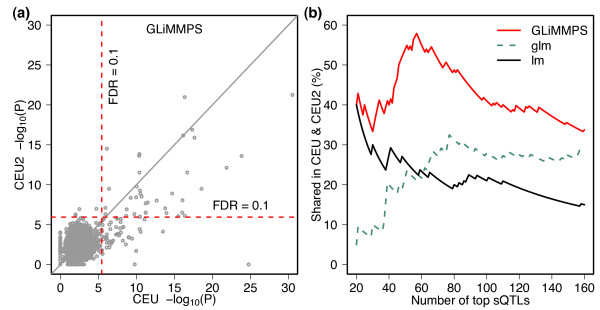
**Concordance of sQTLs in two RNA-seq datasets of the Caucasian (CEU) population as obtained by different statistical models**. **(a) **Comparison of GLiMMPS *P *values for the most significant SNP of each alternatively spliced exon in the CEU and CEU2 datasets. X-axis shows the -log_10_(*P *value) in CEU. Y-axis shows the -log_10_(*P *value) in CEU2. Red lines show the FDR cutoff of 10%. **(b) **Concordance of sQTL rankings between CEU and CEU2 based on different statistical models. The x-axis represents the number of top *n *ranked sQTLs in each dataset, while the y-axis represents the percentage of sQTLs in common between the two datasets among the top *n *sQTLs in CEU, based on *P *value rankings calculated by the linear model (lm), generalized linear model (glm), and GLiMMPS.

To further assess the performance of GLiMMPS, we analyzed the two human RNA-seq datasets on CEU LCL samples (CEU and CEU2) using the GLiMMPS, lm, and glm models. As previous studies suggested that the signal SNPs for most sQTLs are near the target exons [11,16], we carried out sQTL analysis for all common SNPs (minor allele frequency >0.05) within 200 kb from alternatively spliced exons with a median of at least 5 total splice junction reads in both CEU and CEU2 samples. We used permutation to determine the null distribution of minimal *P *values of SNPs near exons. Subsequently we applied the false discovery rate (FDR) correction to establish a cutoff *P *value corresponding to the FDR level of 0.1 (see details in Supplementary Methods and Figure S5 in Additional file 1). This yielded 140 unique AS events in 106 genes with significant sQTL signals in the CEU dataset (Additional file 2). Because of the lower sequencing depth, there were a smaller number (56) of significant sQTLs identified by GLiMMPS in the CEU2 dataset. Nonetheless, the significant sQTL signals identified by GLiMMPS are strongly correlated between the two datasets (Figure 3a). Among the 56 significant sQTLs (FDR ≤0.1) in CEU2, 39 (70%) are also significant in CEU. Although there is a larger proportion of significant sQTLs in CEU showing no significance in CEU2, it is most likely due to the lower sequencing depth in CEU2. To quantitatively compare the relative rankings of sQTLs identified by different models (GLiMMPS, lm, and glm) in CEU and CEU2, we calculated the proportion of sQTL exons among the top *n *most significant in CEU that were also among the top *n *in CEU2 (*n *ranges between 20 and 160). Compared to lm and glm, GLiMMPS produces a much higher concordance of rankings between the two datasets, especially for the top 60 sQTLs which correspond to approximately 10% FDR in the CEU2 dataset (Figure 3b).

To experimentally assess the robustness of GLiMMPS predictions, we randomly selected 24 SE (skipped exon) type and 2 A5SS (alternative 5' splice site) type of sQTLs out of the 140 significant sQTLs detected in the CEU dataset and performed RT-PCR validation using quantitative fluorescent RT-PCR (Materials and methods). For the validation experiments, we used an independent panel of 86 HapMap LCLs covering diverse worldwide populations (Additional file 3). All 26 sQTLs were validated, yielding a validation rate of 100% (Additional file 4; Figure S6 in Additional file 1). In eight individuals analyzed by both RNA-seq and RT-PCR, the exon inclusion levels estimated by RNA-seq were highly correlated with RT-PCR measurements (Pearson correlation coefficient r = 0.87). It is noteworthy to mention that these 26 selected sQTLs have a wide range of *P *value rankings among the 140 significant sQTLs, as opposed to being selected from the top of the significant sQTL list. The interquartile range of their rankings is 38 to 95. This suggests that the vast majority of the sQTLs identified by GLiMMPS represent true signals of splicing variation in human populations.

### GLiMMPS reveals positional features of sQTLs

**Figure 4:**
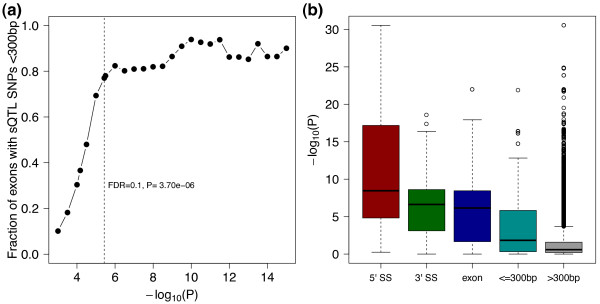
****Positional distribution of sQTL SNPs**. (a) **The fraction of sQTL exons with significant SNPs within 300 bp of the splice sites as a function of the *P *value cutoffs for significant sQTLs. X-axis is the -log_10_(*P *value) for cutoffs to define significant sQTLs. Y-axis is the fraction of sQTL exons with any SNP called significant within 300 bp of the splice sites. **(b) **The boxplot of GLiMMPS *P *values for all SNPs around the 140 significant sQTLs (FDR ≤0.1), grouped into five categories based on the positions of SNPs with respect to the splice sites.

Next we examined the positional distribution of SNPs associated with significant sQTL signals in the CEU dataset. It should be noted that the genotype information for the CEU dataset came from both HapMap and 1000 Genomes project data, thus they capture the vast majority of common SNPs in the human genome. Consistent with previous sQTL studies using arrays and lower-density HapMap SNPs [11,12], sQTL signal SNPs with a GLiMMPS *P *value ≤3.70E-06 (corresponding to FDR ≤0.1) are centered around the splice sites (SS) of target exons. A local examination of the SNP positions for the 140 significant GLiMMPS sQTLs indicates that the precise locations of these SNPs are strongly correlated with their potential impacts on splicing. As we increased the stringency of the *P *value cutoff for significant sQTLs, we observed a steady increase of the proportion of sQTLs with at least one significant signal SNP within 300 bp of the splice sites (Figure 4a). The turning point is around FDR = 0.1, where only around 20% of sQTLs have no significant signal SNPs discovered within 300 bp of the splice sites. To further evaluate the correlation between SNP positions and potential impacts on splicing, we classified all *cis *SNPs within 200 kb of the sQTL exons into five categories according to the SNP location relative to the splice site, where 5' SS represent the nine bases of the 5' splice site including six bases in intron and three bases in exon, and 3' SS represent the 23 bases of the 3' splice site including 20 bases in intron and three bases in exon [23]. We observed a striking difference in the distribution of sQTL *P *values for *cis *SNPs located in different regions (Figure 4b). Specifically, *cis *SNPs located within the 5' SS have the smallest overall *P *values, followed by SNPs within the 3' SS and exons, and intronic SNPs within 300 bp of the splice sites. SNPs located in the distal intronic regions (>300 bp from the splice sites) have the biggest overall sQTL *P *values, suggesting that they are least likely to affect splicing. This trend is consistent with the observation by Pickrell et al. showing the enrichment of sQTL signal SNPs in splice sites [16], but with a finer classification of SNP locations.

To definitively identify causal SNPs underlying significant sQTLs, we tested the effects of individual SNPs on splicing using minigene reporter assays. It should be noted that since multiple SNPs can be in high linkage disequilibrium (LD) with each other, an sQTL signal SNP with high association to exon splicing may not necessarily be the causal SNP that affects splicing regulation. In fact, the 140 significant sQTLs (FDR ≤0.1) have on average 63 significant SNPs. Of the 26 RT-PCR validated sQTLs, the causal SNPs in four genes (*CAST, DHRS1, HMSD*, and *ATP5SL*) were confirmed previously in work by us [24] and others [11]. The causal SNPs in *CAST, DHRS1*, and *HMSD *are located in the 5' SS [24], while the causal SNP in *ATP5SL *is located in the exon and disrupts two putative exonic splicing enhancers [11]. For the remaining RT-PCR confirmed sQTLs, we randomly selected 14 for minigene experiments. Briefly, the target exon and 350-500 bp of surrounding intronic sequences on each side of the exon were sub-cloned into the minigene expression vector and site-directed mutagenesis was carried out to generate the alternative alleles. After transiently transfecting these plasmids into HEK293 cells, we performed quantitative RT-PCR analysis of wild-type and mutant minigene reporters to determine the effect of the SNPs on exon inclusion levels (see details in Materials and methods). In 10 of the 14 exons analyzed (*NTPCR, KIAA1841, SP140, ITM2C, PARP15, PTK2B, BCL2A1, SHMT1, ITPA*, and *ARFGAP3*), the minigene experiments identified at least one SNP that caused >10% change of the minigene exon inclusion levels, with the direction of change matching the RNA-seq/RT-PCR data (Figure S7 in Additional file 1). These include two exons where we found multiple SNPs with additive effects on splicing within one LD block (*KIAA1841*) or multiple LD blocks (*ITPA*). In another two exons (*PPIL3 *and *NCAPG2*), the minigene experiments failed to identify any SNP with strong effect on splicing. For *PPIL3*, the SNP rs111292412 in the 3' SS affected splicing of the minigene reporter in the same direction as in the RNA-seq data, but the change was minor (from 9% in AA to 5% in GG). In *NCAPG2*, the closest sQTL SNP was an intronic SNP 347 bp away from the splice sites, and it did not have any measurable impact on the splicing of the minigene reporter. It is possible that another proximal or distal SNP or indel not genotyped yet is responsible for this sQTL signal. Finally, in the last two exons analyzed (*CLEC2D *and *MX1*), the minigene exon inclusion levels were close to 100% for all alleles, suggesting that the cloned minigene reporters transfected to the HEK293 cells did not faithfully recapitulate endogenous exon splicing activities in the LCLs. Taken together, despite the inherent limitations of minigene reporter systems [25], we were able to use minigene experiments to identify the causal SNPs underlying 10 of the 14 sQTL signals analyzed. In all 10 exons, the causal SNPs confirmed by minigene analysis were located proximal to the alternatively spliced exons (that is, within 300 bp of the splice sites).

### sQTLs explain GWAS signals of human traits and diseases

A powerful application for characterizing human transcriptome variation such as eQTLs and sQTLs is to interpret signals from GWAS studies [26-28]. Although GWAS have had great success in identifying numerous disease susceptibility loci, the peak signal SNPs identified by GWAS provided little information about the underlying causal variants or the molecular mechanisms responsible for the observed association [29]. Compelling evidence indicates that a large fraction of the underlying causal variants affect phenotypes via non-coding (for example, influencing gene regulatory processes such as transcription and RNA processing) as opposed to coding (direct amino acid changes) mechanisms [30,31]. The important role of alternative splicing in shaping the human transcriptome diversity suggests sQTL SNPs may represent the causal variants underlying many observed GWAS signals. Indeed, previous studies of alternative splicing variation using RT-PCR, array, and sequencing based technologies have identified candidate sQTLs linked to GWAS signals [11-13,32-36]. We investigated all significant sQTL SNPs (GLiMMPS FDR ≤0.1) in high (r^2 ^>0.8) linkage disequilibrium (LD) with GWAS signal SNPs listed in the Catalog of Published Genome-Wide Association Studies [37] (see Materials and methods). We identified 10 sQTLs strongly linked to GWAS SNPs of human traits or diseases (Table 1). The list include known splicing altering SNPs for *CAST, ERAP2*, and *ATP5SL*, as well as novel findings with intriguing biological and medical implications.

**Table 1:** The list of sQTL signals linked to GWAS signals.

Gene	AS type^a^	Target exon^b ^(hg19)	sQTL SNP^c^	SNP type	GWAS trait (SNP)	GWAS references
*ACADM*	SE	+chr1:76194085-76194173	rs7524467	< = 300 bp	Metabolic traits (rs211718)	[78]
*DRAM2*	SE	-chr1:111682122-111682288	rs3762374	5' SS	Liver enzyme levels (gamma-glutamyl transferase) (rs1335645)	[79]
*SP140*	SE	+chr2:231110577-231110655	rs28445040	Exon	Chronic lymphocytic leukemia (rs13397985)	[42]
					Multiple sclerosis (rs10201872)	[43]
					Crohn's disease (rs7423615)	[39]
*CAST*	SE	+chr5:96076448-96076487	rs7724759	5' SS	Alcohol dependence (rs13160562)	[38]
*ERAP2*	A5SS^d^	+chr5:96235824-96235949	rs2248374	5' SS	Crohn's disease (rs2549794)	[39]
					Ankylosing spondylitis (rs30187)	[80]
*MRPL11*	A5SS^d^	-chr11:66206102-66206319	rs11110	Exon	Bipolar disorder (rs2242663)	[81]
*ARL6IP4*	A3SS^e^	+chr12:123466117-123466426	rs55742290	3' SS	Platelet counts (rs7296418, rs1727307)	[82]
*ULK3*	MXE^f^	-chr15:75130091-75130139	rs12898397	5' SS	Coffee consumption (rs6495122)	[83]
					Coronary heart disease (rs2472299)	[84]
*ATP5SL*	SE	-chr19:41939176-41939339	rs1043413	Exon	Height (rs17318596)	[41]
*ITPA*	SE	+chr20:3193814-3193872	rs1127354	Exon	Response to hepatitis C treatment (rs11697186, rs6139030)	[50]
					Ribavirin-induced anemia (rs1127354)	[85]

^a^AS type: SE, skipped exon; A5SS, alternative 5' splice site; A3SS, alternative 3' splice site; MXE, mutually exclusive exons.

^b^Exon coordinates are in hg19 with the start position 0 based and the end position 1 based. The direction (+/-) of transcription is denoted before the coordinates.

^c^The significant sQTL SNP (FDR≤0.1) closest to the target exon. SNP position and *P *value from GLiMMPS can be found in Additional file 2.

^d^Alternative 5' SS: ERAP2, chr5:96235893; MRPL11, chr11:66206180.

^e^Alternative 3' SS: ARL6IP4, chr12:123466141.

^f^Mutually exclusive alternative exon: ULK3, chr15:75130492-75130533.

In a previous GWAS study, the SNP rs13160562 near *CAST *was discovered to be significantly associated with alcohol dependence [38]. However, no functional implication of this SNP was discussed in the original study. Here, GLiMMPS identified this SNP as an sQTL signal SNP in *CAST*. It is significantly associated with the splicing of *CAST *exon 13 located 45 kb upstream of the SNP position. It is also in an LD block (r^2 ^= 0.53) with another SNP rs7724759 located in the 5' SS of exon 13, which has been confirmed experimentally to alter the splicing of this exon [11,24]. Thus, genetic variation of alternative splicing is the likely causal mechanism underlying the reported association of *CAST *and alcohol dependence. In *ERAP2*, GLiMMPS identified SNP rs2248374 as an sQTL signal SNP for exon 10. This SNP disrupts the activity of the 5' SS [11]. This sQTL SNP is in high LD (r^2 ^= 0.83) with a GWAS SNP rs2549794, previously identified as significantly associated with Crohn's disease [39]. The skipping of this alternatively spliced exon from *ERAP2 *introduces a premature stop codon, resulting in nonsense-mediated decay of the exon skipping isoform and a dramatic reduction of overall transcript levels, which subsequently impacts antigen representation [40]. Haplotype analysis of the sQTL SNP and its linked SNPs revealed evidence of strong balancing selection during human evolution [40], suggesting the functional and evolutionary importance of this sQTL. A third example is *ATP5SL*, identified as a GWAS locus associated with height in multiple populations [41]. The peak signal SNP reported by GWAS is rs17318596 but the mechanism of this SNP was unclear in the original study. GLiMMPS identified a significant sQTL for exon 5 of *ATP5SL*. The sQTL SNP rs1043413 is strongly linked to the GWAS signal SNP rs17318596 (r^2 ^= 0.84) (Figure S8 in Additional file 1). This sQTL SNP rs1043413 is located in exon 5 and disrupts two exonic splicing enhancers (ESEs) [11]. Together, these data indicate that even at a very modest sequencing depth (28.4-66 million 50 bp single-end reads per individual), GLiMMPS recovered previously reported associations between SNP and splicing that may contribute to phenotypic variation in humans.

**Figure 5:**
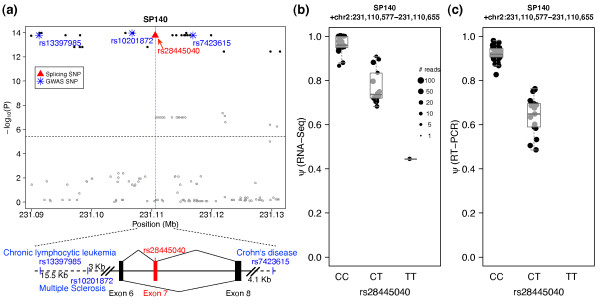
**An example of sQTL signal overlapping with GWAS signal near gene *SP140***. **(a) **The distribution of GLiMMPS *P *values around the sQTL exon (exon 7) in gene *SP140*. The black horizontal dashed line reflects the 10% FDR cutoff and red vertical lines mark the location of the sQTL exon. SNPs in linkage disequilibrium (r^2 ^>0.8 in the CEU population) with the GWAS SNPs (blue asterisks) are shown in solid black dots, while other SNPs are shown in grey circles. The causal splicing SNP in exon 7 is shown in red triangle. Exon-intron structure is shown in the bottom with GWAS SNPs and the causal splicing SNP (rs28445040) marked at corresponding locations. **(b) **Boxplot showing the significant association of rs28445040 with exon inclusion level (*ψ*) of the *SP140 *exon 7 estimated by the CEU RNA-seq dataset. The size of each dot is scaled by the total number of splice junction reads for that individual. **(c) **The same boxplot using exon inclusion level (*ψ*) measured by quantitative RT-PCR.

We also identified novel sQTL signals with interesting functional and disease implications. For example, we identified a novel sQTL signal in *SP140 *associated with previously identified GWAS signals for chronic lymphocytic leukemia [42], multiple sclerosis [43], and Crohn's disease [39]. *SP140 *is a tissue-specific gene whose expression is restricted to lymphoid cells [44]. Its protein domain structure suggests a role in chromatin-mediated regulation of gene expression [45]. A previous GWAS analysis of chronic lymphocytic leukemia identified a risk SNP rs13397985 located in intron 1 of *SP140*. It was proposed that this GWAS signal SNP affects *SP140 *gene transcription [42], but a recent replication study indicates that the association of this SNP to *SP140 *steady state gene expression levels is only marginal (FDR = 0.157 after adjusting for multiple testing) [46]. It should be noted that the difference in gene expression levels among genotype groups is minor and marginal according to the CEU RNA-seq data as well (*P *value = 0.07). On the other hand, GLiMMPS found a novel significant sQTL signal for exon 7 of *SP140 *(Figure 5a). The peak sQTL signal SNP rs28445040 (GLiMMPS *P *value = 1.69E-14) located in exon 7 is in high LD with the GWAS signal SNPs for chronic lymphocytic leukemia (rs13397985, r^2 ^= 1), multiple sclerosis (rs10201872, r^2 ^= 0.92), and Crohn's disease (rs7423615, r^2 ^= 1). The C to T mutation in rs28445040 does not lead to any amino acid change. However, according to RNA-seq data, the average exon inclusion levels for the CC, CT, and TT genotypes were 96%, 77%, and 44%, respectively (Figure 5b). This trend was robustly validated by RT-PCR experiments (Figure 5c). Furthermore, minigene assays confirmed the causal role of rs28445040 in regulating the splicing of *SP140 *exon 7 (Figure S7 in Additional file 1). Collectively, these data strongly suggest that the SNP that alters the splicing of *SP140 *exon 7 is the causal genetic variant responsible for the reported associations with these diseases. The skipping of exon 7 causes an in-frame deletion of a 26 amino acid peptide segment from the SP140 protein product. Interestingly, this peptide segment is located within an intrinsically disordered region as predicted by IUPred [47]. Intrinsically disordered regions are enriched for sites of post-translational modifications and protein-protein interactions, and two recent studies [48,49] show that alternative splicing of exons encoding disordered protein sequences frequently rewires protein-protein interaction networks in the proteome. In the future, it will be interesting to determine how alternative splicing of *SP140 *exon 7 regulates SP140 protein functions and influences downstream cellular phenotypes.

The identification of sQTLs can also help resolve apparent confusions about the causal mechanisms of GWAS signals. For example, the SNP rs11697186 located in gene *DDRGK1 *near the *ITPA *gene (Inosine Triphosphate Pyrophosphohydrolase) was significantly associated with response to hepatitis C treatment in a GWAS study, and later was found to be in high LD with SNP rs1127354 on *ITPA *exon 2 by fine mapping [50]. Of note, this C-to-A SNP (rs1127354) on exon 2 has been well established in the pharmacogenetics field to be associated with ITPA enzyme deficiency or low-activity [51,52], but the molecular mechanism was unclear. This non-synonymous SNP causes a proline to threonine change (P32T) in the IPTA protein product. However, based on the crystal structure of the human ITPA protein, the proline residue was far away from the active site of the enzyme [53]. Moreover, recent biochemical studies of ITPA showed that the purified mutant protein with the P32T change has the same activity as the wild-type protein [54]. Others have proposed the alternative mechanism that this exon 2 SNP causes mis-splicing of *ITPA *[55], but the properties of the gene product resulting from mis-splicing have not been examined. Our analysis of the CEU RNA-seq data identified the same *ITPA *exon 2 SNP as a significant sQTL signal SNP (*P *value = 5.80E-09) associated with the combined skipping of exons 2 and 3. This prediction is robustly validated by RT-PCR (Figure S9 in Additional file 1). Minigene experiments further confirmed that this exonic SNP as well as an adjacent intronic SNP (rs7270101) both reduced the inclusion levels of exons 2 and 3 (Figure S7 in Additional file 1). These results reinforce the proposed effect of this *ITPA *SNP at the RNA level [55], and suggest that future studies on the causal mechanism of this *ITPA *gene variant should compare the activities of the full-length protein isoform to the truncated isoform that lacks exons 2 and 3.

## Discussion

We have developed GLiMMPS, a generalized linear mixed model to detect genotype-splicing associations from RNA-seq data. The key advantage of GLiMMPS over previously used methods is that it models: (1) variation in exon-specific read coverage across individuals; and (2) overdispersion in RNA-seq read counts. Both issues are important for accurate exon-level expression quantitation. The coverage of RNA-seq reads for any given alternative exon is a critical factor for the precision of the exon inclusion level estimate [14,56]. The importance of accounting for overdispersion in RNA-seq data analysis has also been well recognized [57]. Methods based on the negative binomial model [58,59] or the generalized linear model with Cox-Reid dispersion estimators [19,20] have been developed for modeling dispersion in detecting differential gene or exon expression between biological states. Here in the sQTLs analysis, by modeling these two levels of variation in RNA-seq read counts, GLiMMPS achieves superior performance over competing statistical models, as demonstrated by analyses of simulated and real RNA-seq data. Importantly, even at a low coverage we observed a high level of concordance in the GLiMMPS results between the two human datasets (CEU and CEU2). Additionally, RT-PCR tests of 26 randomly selected significant sQTLs yielded a validation rate of 100%. Together, these results demonstrate that GLiMMPS is a robust and improved method to detect sQTLs from RNA-seq data.

Fine-scale analysis of sQTLs reveals positional features of SNPs that alter exon splicing. We found that the location of the SNPs is strongly correlated with potential impact on splicing (Figure 4b). Specifically, SNPs located within the 5' and 3' splice sites have the smallest (most significant) overall GLiMMPS *P *values, consistent with the importance of the splice sites in exon recognition during pre-mRNA splicing. Interestingly, the significance level of sQTLs is positively correlated with the proximity of the sQTL signal SNPs to target exons. As we increased the significance level cutoff for sQTLs, we observed a progressive increase of the proportion of sQTLs with at least one significant signal SNP within 300 bp of the splice sites (Figure 4a). The causal roles of these proximal sQTL SNPs on exon splicing were further confirmed by minigene splicing reporter assays. Collectively, these results support the hypothesis that the majority of *cis *regulatory information controlling alternative splicing is encoded in close proximity (for example, within 300 bp) of the target exons, consistent with a recent analysis of the mammalian splicing code [60]. Nonetheless, it should also be noted that 20% of the significant sQTLs (FDR ≤0.1) lack any significant signal SNP within 300 bp of the splice sites, including sQTLs confirmed experimentally by RT-PCR (in *NCAPG2 *and *PIGQ*, see Figure S6 in Additional file 1). For such sQTLs, it is possible that the causal SNPs are indeed proximal, but are missing from current SNP annotations or fail to reach the significance level cutoff due to small sample size. Alternatively, we cannot rule out the possibility that a small fraction of sQTLs are indeed due to SNPs disrupting distal splicing regulatory elements, given that the physical binding sites of splicing factors on the pre-mRNA can be located deep into the introns [61]. In the future, it would be interesting to confirm the identity and elucidate the regulatory mechanisms of causal sQTL SNPs acting in introns distal to target exons.

The detection of sQTLs is useful for interpreting signals from GWAS studies. Despite the success of GWAS in revealing the genetic basis of complex traits and diseases, elucidating the mechanistic implications of GWAS findings remains a major challenge [29]. As many functional SNPs may affect gene expression and regulation instead of the final protein sequence, integrating transcriptome information with GWAS signals has proven to be an effective approach for pinpointing the functional causal variants underlying GWAS signals [62-64]. Here, from the CEU RNA-seq dataset we identified 140 unique sQTLs, including 10 significantly linked to previously identified GWAS signals (Table 1). This is probably only scratching the surface of trait-associated sQTLs, due to the low sequencing depth (28.4-66 million single-end reads per individual) and the small sample size (41 individuals). We anticipate that with more and deeper RNA-seq data generated for diverse human tissues and cell types, the catalog of sQTLs linked to phenotypic traits and diseases will rapidly expand in the near future.

The GLiMMPS framework provides the basis for several aspects of future extensions. Currently, GLiMMPS uses reads mapped to splice junctions to estimate exon inclusion levels. This is a commonly used approach in alternative splicing quantitation from RNA-seq data [56,65,66]. However, with proper normalization for lengths of isoform-specific segments, it is feasible to also incorporate reads mapped within the exons, which may further improve the power in detecting sQTLs. This could be particularly useful for strand-specific RNA-seq, where the origins of exon body reads can be unambiguously assigned to sense or antisense transcripts. Additionally, in paired-end RNA-seq data with tight distribution of insert size, reads that map to flanking constitutive exons can also provide useful information about the exon inclusion level [14]. Furthermore, RNA-seq reads often display non-uniform distribution along mRNA transcripts due to sequence-specific bias in RNA sequencing, and several methods have been developed to model and correct for such biases [67-70]. In principle, we can use a suitable bias correction method to adjust the raw RNA-seq read counts, prior to analysis by GLiMMPS. However, we tested two well-known bias correction methods [67,68] using a deep RNA-seq dataset with matching quantitative RT-PCR data for over 100 exons in two cell lines [66,71], but did not observe improvement in the RNA-seq estimates of exon inclusion level as judged by the correlation of RNA-seq estimates with the RT-PCR measurements. Another area of improvement is to consider the potential impact of specific SNPs on exon splicing as the prior in the statistical model, an idea previously used for detecting expression QTLs [72-74]. For example, our results show a significant association between the SNP position and the potential impact on splicing (Figure 4), with SNPs located in the 5' and 3' splice sites most likely to influence exon splicing. It is possible to incorporate such positional information or more advanced predictive models of exon splicing [60] as the prior information to guide the detection of sQTLs.

## Conclusions

RNA-seq has become a powerful and increasingly affordable technology for population-scale analysis of transcriptome variation. Here we report GLiMMPS, a robust statistical method for detecting splicing quantitative trait loci (sQTLs) from RNA-seq data. GLiMMPS is applicable to all major patterns of alternative splicing events. The GLiMMPS source code and user manual are freely available for download at [75]. As the cost of high-throughput sequencing continues to decline, we anticipate that combined sequencing of genomes and transcriptomes will become a popular design in large-scale studies of traits and diseases. GLiMMPS provides a useful tool for genome-wide identification of sQTLs from population-scale RNA-seq datasets.

## Materials and methods

### RNA-seq datasets

We downloaded the RNA-seq datasets produced by [18] and [17]. Both datasets came from the lymphoblastoid B cell lines from the Caucasian (CEU) population in the HapMap project [8]. There were 28.4-66 million 50 bp single end reads sequenced for 41 individuals by Cheung et al., while there were only 3.5-17.1 million 37 bp paired end reads for 60 individuals by Montgomery et al. We denote the datasets from Cheung et al. and Montgomery et al. as CEU and CEU2, respectively. Because the sequencing depth in CEU is much higher than in CEU2, we focused our analysis on the CEU dataset, but also carried out comparison between CEU and CEU2. RNA-seq sequence reads were mapped to the reference human genome (hg19) using Tophat [21] with Ensembl gene annotations (Ensembl genes r65). The CEU and CEU2 datasets were mapped with the single end or the paired end mode respectively. Only uniquely mapped reads were retained for downstream analysis.

To search for sQTLs, we first identified all alternative splicing events and RNA-seq reads mapped to splice junctions using the MATS pipeline as described previously [66]. We then focused our analysis on four types of alternative splicing events: skipped exon (SE), alternative 5' splice site (A5SS), alternative 3' splice site (A3SS), and mutually exclusive exons (MXE). Using splice junction reads, we can obtain a point estimate of the exon inclusion level (*ψ*). Given that we have an observed number of splice junction reads for one isoform (*y*) and total splice junction read counts (*n*), then ψ=y/n (see Figure S1 in Additional file 1). We then filtered out exons with no or little change in exon inclusion level (|Δψ|<0.1) or few total junction read counts (median *n *<5) in the population, and obtained 18,267 AS events from CEU and 7,747 AS events from CEU2 for the downstream sQTL analysis.

### Genotype data

The genotype data for the 41 individuals in the CEU dataset were taken from the latest HapMap3 release (#28). Of these 41 individuals, 23 were also genotyped in the 1000 Genomes project [9]. For SNPs uniquely reported by the 1000 Genomes project, we imputed the genotypes for individuals not in the 1000 Genomes project using Beagle [76]. We filtered out low frequency SNPs with MAF (minor allele frequency) <0.05. For each alternatively spliced exon, we tested *cis *SNPs within 200 kb upstream or downstream of the target exon splice sites when searching for sQTLs. For the CEU2 dataset, the 60 individuals were all included in the 1000 Genomes project. Fifty-eight of them were sequenced in low coverage and two were in high coverage. To avoid genotype calling bias, we only included the 58 low-coverage individuals with genotypes taken directly from the 1000 Genomes project data (10/2010 release). The same MAF filtering was used as in the CEU dataset.

### Statistical models for sQTL analysis

All statistical analyses were done in the R statistical environment [77]. We evaluated three different models for sQTL analysis: linear model (lm), generalized linear model (glm), and our proposed generalized linear mixed model (GLiMMPS). The model details were provided in Supplementary Methods in Additional file 1. Here we only briefly describe the GLIMMPS model. GLiMMPS is a hierarchical model that uses the reads information from both exon inclusion and skipping isoforms instead of only a point estimate of exon inclusion level (as in the lm model used in [16,17]) in sQTL analysis. Given the observed junction read counts as in Figure S1 in Additional file 1 we assume that these junction reads supporting two alternative isoforms follow a binomial distribution: $y_{i}|\psi_{i}\sim Binomial\left(n_{i},\psi_{i}\right)$. To deal with the overdispersion in the generalized linear model, we model the extra variance of *ψ *as a random effect for each individual *i *in the regression model with random effects, $u_{ij}\sim N\left(0,\sigma_{uj}^{2}\right)$[22]. Let $u_{ij}=\sigma_{uj}z_{ij}$, where $z_{ij}\sim N\left(0,1\right)$, *β_j _*denoting the fixed effect for SNP *j*, the second level of the model can be written as: $\psi_{i}=\text{log}it^{-1}\left(\beta_{0}+\beta_{j}g_{ij}+\sigma_{uj}z_{ij}\right)$. Thus the joint likelihood for *β*, $\sigma_{uj}^{}$ is given by:

$L\left(\beta ,\sigma_{uj}\right)=\overset{m}{\underset{i=1}{\prod }}\left(\begin{array}{c} n_{i}\\ y_{i} \end{array}\right)\int \frac{exp{\left\{\left(\beta_{0}+\beta_{j}g_{ij}+\sigma_{uj}z_{ij}\right)\right\}}^{y_{i}}}{{\left[1+exp\left\{\left(\beta_{0}+\beta_{j}g_{ij}+\sigma_{uj}z_{ij}\right)\right\}\right]}^{n_{i}}}N\left(z_{ij}\right)dz_{ij}$,

where N(⋅) is the standard normal density. Function glmer() from R package lme4 was used to fit the model, where Laplace approximation is used for the parameter estimations and a likelihood ratio test was used to obtain the *P *values for the fixed effect *β_j _*for each SNP *j*.

For both the CEU and CEU2 datasets, using each of the statistical models (lm, glm, and GLiMMPS) mentioned above, we carried out the analysis for each exon with SNPs within 200 kb of the exon. To estimate the false discovery rate, we used the same permutation approach as in [16] to obtain the null distribution of the *P *values. The details are in Supplementary Methods in Additional file 1.

### RT-PCR validation

To validate the sQTLs found in the CEU datasets, we randomly selected 26 significant sQTL exons (FDR ≤0.1) for RT-PCR validation. We performed the validation experiments on an independent panel of 86 lymphoblastoid cell lines from the HapMap3 project (Additional file 3), which were purchased from the Coriell Institute for Medical Research, Camden, NJ, USA. Total RNA was extracted using TRIzol (Invitrogen, Carlsbad, CA, USA) and reverse transcribed by the High-Capacity cDNA Reverse Transcription Kit (Applied Biosystems, Foster City, CA, USA). Fluorescently labeled RT-PCR was carried out as described before [24]. Capillary electrophoresis (Georgia Genomics Facility, Athens, GA, USA) and 5% Urea TBE-PAGE were used for resolving PCR products. In capillary electrophoresis, band peak area was generated by GeneMapper 4.0 software (Applied Biosystems, Carlsbad, CA, USA). In 5% Urea PAGE, the signal was captured by Fujifilm FLA-7000 (Fuji Photo Film Co. Ltd., Tokyo, Japan) and quantified using the ImageQuant TL7.0 software (General Electric Company, Waukesha, WI, USA). Final exon inclusion level was calculated as the peak area or band intensity of the exon inclusion band(s) divided by the total peak areas or band intensities of all bands. To test the association of genotypes with the RT-PCR estimated exon inclusion levels, we used the most significant HapMap3 sQTL SNP for each target exon. A linear regression on the estimated exon inclusion levels with the SNP genotypes of the SNP was used to calculate *P *values and those with *P *value <0.05 were called as validated. All RT-PCR primer sequences are listed in Additional file 4 and individual exon inclusion levels are listed in Additional file 5.

### Minigene analysis

We used the hybrid construct pI-11-H3 (provided by Dr. Russ P. Carstens, University of Pennsylvania, Philadelphia, PA, USA) for our minigene splicing reporter assays. Genomic DNAs were extracted from LCLs using UltraClean™ Tissue&Cells DNA Isolation kit (MO BIO Laboratories, Carlsbad, CA, USA). The target exon and its flanking 350-500 bp intronic regions were amplified by PCR (see Additional file 6 for the primer sequences). In-Fusion™ Advantage PCR Cloning Kit (Clontech, Mountain View, CA, USA) or restriction enzyme digestion and ligation strategy were used to clone PCR products into the vector. Site-directed mutagenesis was carried out following the manufacturer's instructions. The integrity of all constructs was confirmed by sequencing. To test minigene splicing, plasmids were transiently transfected into HEK293 cells. Fluorescently labeled RT-PCR was performed to evaluate the splicing impact of specific polymorphisms as described before [24].

### GWAS signals

We obtained 7,523 GWAS SNPs at genome-wide significance level of *P *value <10^-5 ^from the Catalog of Published Genome-Wide Association Studies (accessed 03/30/2012) [37]. Using all the 1000 Genomes SNPs from the CEU population, we obtained all SNPs that are in high linkage disequilibrium with the GWAS SNPs (r^2^>0.8 in the CEU population and within 200 kb window of the GWAS SNP). Because of the high SNP density and high recombination rate around the MHC region, we excluded genes from this region in this part of the analysis. We then identified sQTL signal SNPs overlapped with this expanded list of GWAS linked SNPs.

### Data access and source code availability

The GLiMMPS model has been implemented and released in an easy to use package. The splice junction read counts, genotypes, and validation datasets, as well as the source code used for sQTL processing and analysis are provided at the companion website of this article [75].

## Abbreviations

A3SS, alternative 3' splice site; A5SS, alternative 5' splice site; AS, alternative splicing; CEU, Utah residents of European descent from CEPH-Centre d'Etude du Polymporphisme Humain; CV, coefficient of variation; GLiMMPS, Generalized Linear Mixed Model Prediction of sQTL; glm, generalized linear model; lm, linear model; LCL, lymphoblastoid B cell line; MAF, minor allele frequency; MXE, mutually exclusive exons; QTL, Quantitative Trait Loci; RNA-seq, RNA sequencing; ROC, receiver operating characteristic; SE, skipped exon; SNP, single-nucleotide polymorphism; sQTL, splicing Quantitative Trait Loci; SS, splice site.

## Competing interests

All authors declare that they have no competing interests.

## Authors' contributions

KZ and YX designed the project; KZ developed the algorithm; KZ, ZXL, and JWP carried out all the experiments; KZ, QZ, and YX wrote the manuscript. All authors have read and approved the final manuscript.

## Supplementary Material

Additional file 1 **Supplementary Methods and Supplementary Figures S1-S9**. Click here for file

Additional file 2 **Supplementary table S1**. sQTL exons (FDR ≤0.1) and information of the most proximal sQTL SNPs. Click here for file

Additional file 3 **Supplementary table S2**. HapMap3 samples used for RT-PCR validation of sQTLs. Click here for file

Additional file 4 **Supplementary table S3**. Primers and RT-PCR results for validation of sQTLs. Click here for file

Additional file 5 **Supplementary table S4**. The individual exon inclusion levels for RT-PCR validation of sQTLs. Click here for file

Additional file 6 **Supplementary table S5**. Primers used for constructing minigene splicing reporters. Click here for file

## References

[B1] Nilsen TW Graveley BR Expansion of the eukaryotic proteome by alternative splicing. Nature 2010 14 457 463 10.1038/nature08909 20110989 PMC3443858

[B2] Wang ET Sandberg R Luo S Khrebtukova I Zhang L Mayr C Kingsmore SF Schroth GP Burge CB Alternative isoform regulation in human tissue transcriptomes. Nature 2008 14 470 476 10.1038/nature07509 18978772 PMC2593745

[B3] Faustino NA Cooper TA Pre-mRNA splicing and human disease. Genes Dev 2003 14 419 437 10.1101/gad.1048803 12600935

[B4] Wang GS Cooper TA Splicing in disease: disruption of the splicing code and the decoding machinery. Nat Rev Genet 2007 14 749 761 10.1038/nrg2164 17726481

[B5] Wang Z Burge CB Splicing regulation: from a parts list of regulatory elements to an integrated splicing code. RNA 2008 14 802 813 10.1261/rna.876308 18369186 PMC2327353

[B6] Lu Z-X Jiang P Xing Y Genetic variation of pre-mRNA alternative splicing in human populations. Wiley Interdisciplinary Reviews RNA 2012 14 581 592 10.1002/wrna.120 22095823 PMC3339278

[B7] Heinzen EL Yoon W Tate SK Sen A Wood NW Sisodiya SM Goldstein DB Nova2 interacts with a cis-acting polymorphism to influence the proportions of drug-responsive splice variants of SCN1A. Am J Hum Genet 2007 14 876 883 10.1086/516650 17436242 PMC1852745

[B8] Altshuler DM Gibbs RA Peltonen L Altshuler DM Gibbs RA Peltonen L Dermitzakis E Schaffner SF Yu F Peltonen L Dermitzakis E Bonnen PE Altshuler DM Gibbs RA de Bakker PI Deloukas P Gabriel SB Gwilliam R Hunt S Inouye M Jia X Palotie A Parkin M Whittaker P Yu F Chang K Hawes A Lewis LR Ren Y Wheeler D Integrating common and rare genetic variation in diverse human populations. Nature 2010 14 52 58 10.1038/nature09298 20811451 PMC3173859

[B9] Consortium TGP A map of human genome variation from population-scale sequencing. Nature 2010 14 1061 1073 10.1038/nature09534 20981092 PMC3042601

[B10] Kwan T Benovoy D Dias C Gurd S Provencher C Beaulieu P Hudson TJ Sladek R Majewski J Genome-wide analysis of transcript isoform variation in humans. Nat Genet 2008 14 225 231 10.1038/ng.2007.57 18193047

[B11] Coulombe-Huntington J Lam KCL Dias C Majewski J Fine-scale variation and genetic determinants of alternative splicing across individuals. PLoS Genet 2009 14 e1000766 10.1371/journal.pgen.1000766 20011102 PMC2780703

[B12] Fraser HB Xie X Common polymorphic transcript variation in human disease. Genome Res 2009 14 567 575 10.1101/gr.083477.108 19189928

[B13] Heinzen EL Ge D Cronin KD Maia JM Shianna KV Gabriel WN Welsh-Bohmer KA Hulette CM Denny TN Goldstein DB Tissue-specific genetic control of splicing: implications for the study of complex traits. PLoS Biol 2008 14 e1 19222302 10.1371/journal.pbio.1000001 PMC2605930

[B14] Katz Y Wang ET Airoldi EM Burge CB Analysis and design of RNA sequencing experiments for identifying isoform regulation. Nat Methods 2010 14 1009 1015 10.1038/nmeth.1528 21057496 PMC3037023

[B15] Benovoy D Kwan T Majewski J Effect of polymorphisms within probe-target sequences on olignonucleotide microarray experiments. Nucleic Acids Res 2008 14 4417 4423 10.1093/nar/gkn409 18596082 PMC2490733

[B16] Pickrell J Marioni J Pai A Degner J Engelhardt B Nkadori E Veyrieras J-B Stephens M Gilad Y Pritchard J Understanding mechanisms underlying human gene expression variation with RNA sequencing. Nature 2010 14 768 772 10.1038/nature08872 20220758 PMC3089435

[B17] Montgomery S Sammeth M Gutierrez-Arcelus M Lach R Ingle C Nisbett J Guigo R Dermitzakis E Transcriptome genetics using second generation sequencing in a Caucasian population. Nature 2010 14 773 777 10.1038/nature08903 20220756 PMC3836232

[B18] Cheung VG Nayak RR Wang IX Elwyn S Cousins SM Morley M Spielman RS Polymorphic cis- and trans-regulation of human gene expression. PLoS Biol 2010 14 e1000480 10.1371/journal.pbio.1000480 20856902 PMC2939022

[B19] Anders S Reyes A Huber W Detecting differential usage of exons from RNA-seq data. Genome Res 2012 14 2008 2017 10.1101/gr.133744.111 22722343 PMC3460195

[B20] McCarthy DJ Chen Y Smyth GK Differential expression analysis of multifactor RNA-Seq experiments with respect to biological variation. Nucleic Acids Res 2012 14 4288 4297 10.1093/nar/gks042 22287627 PMC3378882

[B21] Trapnell C Pachter L Salzberg SL TopHat: discovering splice junctions with RNA-Seq. Bioinformatics 2009 14 1105 1111 10.1093/bioinformatics/btp120 19289445 PMC2672628

[B22] Browne WJ Subramanian SV Jones K Goldstein H Variance partitioning in multilevel logistic models that exhibit overdispersion. J Roy Stat Soc a Sta 2005 14 599 613 10.1111/j.1467-985X.2004.00365.x

[B23] Yeo G Burge CB Maximum entropy modeling of short sequence motifs with applications to RNA splicing signals. J Comput Biol 2004 14 377 394 10.1089/1066527041410418 15285897

[B24] Lu ZX Jiang P Cai JJ Xing Y Context-dependent robustness to 5' splice site polymorphisms in human populations. Hum Mol Genet 2011 14 1084 1096 10.1093/hmg/ddq553 21224255 PMC3043661

[B25] Singh G Cooper TA Minigene reporter for identification and analysis of cis elements and trans factors affecting pre-mRNA splicing. Biotechniques 2006 14 177 181 10.2144/000112208 16925019

[B26] Schadt EE Molony C Chudin E Hao K Yang X Lum PY Kasarskis A Zhang B Wang S Suver C Zhu J Millstein J Sieberts S Lamb J Guhathakurta D Derry J Storey JD Avila-Campillo I Kruger MJ Johnson JM Rohl CA van Nas A Mehrabian M Drake TA Lusis AJ Smith RC Guengerich FP Strom SC Schuetz E Rushmore TH Mapping the genetic architecture of gene expression in human liver. PLoS Biol 2008 14 e107 10.1371/journal.pbio.0060107 18462017 PMC2365981

[B27] Nicolae DL Gamazon E Zhang W Duan SW Dolan ME Cox NJ Trait-associated SNPs are more likely to be eQTLs: annotation to enhance discovery from GWAS. PLoS Genet 2010 14 e1000888 10.1371/journal.pgen.1000888 20369019 PMC2848547

[B28] Boyle AP Hong EL Hariharan M Cheng Y Schaub MA Kasowski M Karczewski KJ Park J Hitz BC Weng S Cherry JM Snyder M Annotation of functional variation in personal genomes using RegulomeDB. Genome Res 2012 14 1790 1797 10.1101/gr.137323.112 22955989 PMC3431494

[B29] Ioannidis JP Thomas G Daly MJ Validating, augmenting and refining genome-wide association signals. Nat Rev Genet 2009 14 318 329 19373277 10.1038/nrg2544 PMC7877552

[B30] Saccone SF Bolze R Thomas P Quan JX Mehta G Deelman E Tischfield JA Rice JP SPOT: a web-based tool for using biological databases to prioritize SNPs after a genome-wide association study. Nucleic Acids Res 2010 14 W201 W209 10.1093/nar/gkq513 20529875 PMC2896195

[B31] Schaub MA Boyle AP Kundaje A Batzoglou S Snyder M Linking disease associations with regulatory information in the human genome. Genome Res 2012 14 1748 1759 10.1101/gr.136127.111 22955986 PMC3431491

[B32] Xu ZL Taylor JA SNPinfo: integrating GWAS and candidate gene information into functional SNP selection for genetic association studies. Nucleic Acids Res 2009 14 W600 W605 10.1093/nar/gkp290 19417063 PMC2703930

[B33] Need AC Ge DL Weale ME Maia J Feng S Heinzen EL Shianna KV Yoon W Kasperaviciute D Gennarelli M Strittmatter WJ Bonvicini C Rossi G Jayathilake K Cola PA McEvoy JP Keefe RSE Fisher EMC St Jean PL Giegling I Hartmann AM Moller HJ Ruppert A Fraser G Crombie C Middleton LT St Clair D Roses AD Muglia P Francks C A genome-wide investigation of SNPs and CNVs in schizophrenia. PLoS Genet 2009 14 e1000373 10.1371/journal.pgen.1000373 19197363 PMC2631150

[B34] Li G Bahn JH Lee JH Peng GD Chen ZG Nelson SF Xiao XS Identification of allele-specific alternative mRNA processing via transcriptome sequencing. Nucleic Acids Res 2012 14 e104 10.1093/nar/gks280 22467206 PMC3401465

[B35] Hull J Campino S Rowlands K Chan M-S Copley RR Taylor MS Rockett K Elvidge G Keating B Knight J Kwiatkowski D Identification of common genetic variation that modulates alternative splicing. PLoS Genet 2007 14 e99 10.1371/journal.pgen.0030099 17571926 PMC1904363

[B36] Lee Y Gamazon ER Rebman E Lee Y Lee S Dolan ME Cox NJ Lussier YA Variants affecting exon skipping contribute to complex traits. PLoS Genet 2012 14 e1002998 10.1371/journal.pgen.1002998 23133393 PMC3486879

[B37] Hindorff LA Sethupathy P Junkins HA Ramos EM Mehta JP Collins FS Manolio TA Potential etiologic and functional implications of genome-wide association loci for human diseases and traits. Proc Natl Acad Sci USA 2009 14 9362 9367 10.1073/pnas.0903103106 19474294 PMC2687147

[B38] Treutlein J Cichon S Ridinger M Wodarz N Soyka M Zill P Maier W Moessner R Gaebel W Dahmen N Fehr C Scherbaum N Steffens M Ludwig KU Frank J Wichmann HE Schreiber S Dragano N Sommer WH Leonardi-Essmann F Lourdusamy A Gebicke-Haerter P Wienker TF Sullivan PF Nothen MM Kiefer F Spanagel R Mann K Rietschel M Genome-wide association study of alcohol dependence. Arch Gen Psychiatry 2009 14 773 784 10.1001/archgenpsychiatry.2009.83 19581569 PMC4229246

[B39] Franke A McGovern DP Barrett JC Wang K Radford-Smith GL Ahmad T Lees CW Balschun T Lee J Roberts R Anderson CA Bis JC Bumpstead S Ellinghaus D Festen EM Georges M Green T Haritunians T Jostins L Latiano A Mathew CG Montgomery GW Prescott NJ Raychaudhuri S Rotter JI Schumm P Sharma Y Simms LA Taylor KD Whiteman D Genome-wide meta-analysis increases to 71 the number of confirmed Crohn's disease susceptibility loci. Nat Genet 2010 14 1118 1125 10.1038/ng.717 21102463 PMC3299551

[B40] Andres AM Dennis MY Kretzschmar WW Cannons JL Lee-Lin SQ Hurle B Schwartzberg PL Williamson SH Bustamante CD Nielsen R Clark AG Green ED Balancing selection maintains a form of ERAP2 that undergoes nonsense-mediated decay and affects antigen presentation. PLoS Genet 2010 14 e1001157 10.1371/journal.pgen.1001157 20976248 PMC2954825

[B41] Lango Allen H Estrada K Lettre G Berndt SI Weedon MN Rivadeneira F Willer CJ Jackson AU Vedantam S Raychaudhuri S Ferreira T Wood AR Weyant RJ Segre AV Speliotes EK Wheeler E Soranzo N Park JH Yang J Gudbjartsson D Heard-Costa NL Randall JC Qi L Vernon Smith A Magi R Pastinen T Liang L Heid IM Luan J Thorleifsson G Hundreds of variants clustered in genomic loci and biological pathways affect human height. Nature 2010 14 832 838 10.1038/nature09410 20881960 PMC2955183

[B42] Di Bernardo MC Crowther-Swanepoel D Broderick P Webb E Sellick G Wild R Sullivan K Vijayakrishnan J Wang Y Pittman AM Sunter NJ Hall AG Dyer MJ Matutes E Dearden C Mainou-Fowler T Jackson GH Summerfield G Harris RJ Pettitt AR Hillmen P Allsup DJ Bailey JR Pratt G Pepper C Fegan C Allan JM Catovsky D Houlston RS A genome-wide association study identifies six susceptibility loci for chronic lymphocytic leukemia. Nat Genet 2008 14 1204 1210 10.1038/ng.219 18758461

[B43] Sawcer S Hellenthal G Pirinen M Spencer CC Patsopoulos NA Moutsianas L Dilthey A Su Z Freeman C Hunt SE Edkins S Gray E Booth DR Potter SC Goris A Band G Oturai AB Strange A Saarela J Bellenguez C Fontaine B Gillman M Hemmer B Gwilliam R Zipp F Jayakumar A Martin R Leslie S Hawkins S Giannoulatou E Genetic risk and a primary role for cell-mediated immune mechanisms in multiple sclerosis. Nature 2011 14 214 219 10.1038/nature10251 21833088 PMC3182531

[B44] Bloch DB de la Monte SM Guigaouri P Filippov A Bloch KD Identification and characterization of a leukocyte-specific component of the nuclear body. J Biol Chem 1996 14 29198 29204 10.1074/jbc.271.46.29198 8910577

[B45] Dent AL Yewdell J Puvion-Dutilleul F Koken MH de The H Staudt LM LYSP100-associated nuclear domains (LANDs): description of a new class of subnuclear structures and their relationship to PML nuclear bodies. Blood 1996 14 1423 1426 8695863

[B46] Sille FC Thomas R Smith MT Conde L Skibola CF Post-GWAS functional characterization of susceptibility variants for chronic lymphocytic leukemia. PLoS ONE 2012 14 e29632 10.1371/journal.pone.0029632 22235315 PMC3250464

[B47] Dosztanyi Z Csizmok V Tompa P Simon I IUPred: web server for the prediction of intrinsically unstructured regions of proteins based on estimated energy content. Bioinformatics 2005 14 3433 3434 10.1093/bioinformatics/bti541 15955779

[B48] Ellis JD Barrios-Rodiles M Colak R Irimia M Kim T Calarco JA Wang X Pan Q O'Hanlon D Kim PM Wrana JL Blencowe BJ Tissue-specific alternative splicing remodels protein-protein interaction networks. Mol Cell 2012 14 884 892 10.1016/j.molcel.2012.05.037 22749401

[B49] Buljan M Chalancon G Eustermann S Wagner GP Fuxreiter M Bateman A Babu MM Tissue-specific splicing of disordered segments that embed binding motifs rewires protein interaction networks. Mol Cell 2012 14 871 883 10.1016/j.molcel.2012.05.039 22749400 PMC3437557

[B50] Tanaka Y Kurosaki M Nishida N Sugiyama M Matsuura K Sakamoto N Enomoto N Yatsuhashi H Nishiguchi S Hino K Hige S Itoh Y Tanaka E Mochida S Honda M Hiasa Y Koike A Sugauchi F Kaneko S Izumi N Tokunaga K Mizokami M Genome-wide association study identified ITPA/DDRGK1 variants reflecting thrombocytopenia in pegylated interferon and ribavirin therapy for chronic hepatitis C. Hum Mol Genet 2011 14 3507 3516 10.1093/hmg/ddr249 21659334

[B51] Sumi S Marinaki AM Arenas M Fairbanks L Shobowale-Bakre M Rees DC Thein SL Ansari A Sanderson J De Abreu RA Simmonds HA Duley JA Genetic basis of inosine triphosphate pyrophosphohydrolase deficiency. Hum Genet 2002 14 360 367 10.1007/s00439-002-0798-z 12384777

[B52] Maeda T Sumi S Ueta A Ohkubo Y Ito T Marinaki AM Kurono Y Hasegawa S Togari H Genetic basis of inosine triphosphate pyrophosphohydrolase deficiency in the Japanese population. Mol Genet Metab 2005 14 271 279 10.1016/j.ymgme.2005.03.011 15946879

[B53] Stenmark P Kursula P Flodin S Graslund S Landry R Nordlund P Schuler H Crystal structure of human inosine triphosphatase. Substrate binding and implication of the inosine triphosphatase deficiency mutation P32T. J Biol Chem 2007 14 3182 3187 17138556 10.1074/jbc.M609838200

[B54] Stepchenkova EI Tarakhovskaya ER Spitler K Frahm C Menezes MR Simone PD Kolar C Marky LA Borgstahl GE Pavlov YI Functional study of the P32T ITPA variant associated with drug sensitivity in humans. J Mol Biol 2009 14 602 613 10.1016/j.jmb.2009.07.051 19631656 PMC2745931

[B55] Arenas M Duley J Sumi S Sanderson J Marinaki A The ITPA c.94C>A and g.IVS2+21A>C sequence variants contribute to missplicing of the ITPA gene. Biochim Biophys Acta 2007 14 96 102 10.1016/j.bbadis.2006.10.006 17113761

[B56] Pan Q Shai O Lee LJ Frey BJ Blencowe BJ Deep surveying of alternative splicing complexity in the human transcriptome by high-throughput sequencing. Nat Genet 2008 14 1413 1415 10.1038/ng.259 18978789

[B57] Hansen KD Wu Z Irizarry RA Leek JT Sequencing technology does not eliminate biological variability. Nat Biotechnol 2011 14 572 573 10.1038/nbt.1910 21747377 PMC3137276

[B58] Robinson MD Smyth GK Small-sample estimation of negative binomial dispersion, with applications to SAGE data. Biostatistics 2008 14 321 332 17728317 10.1093/biostatistics/kxm030

[B59] Robinson MD McCarthy DJ Smyth GK edgeR: a Bioconductor package for differential expression analysis of digital gene expression data. Bioinformatics 2010 14 139 140 10.1093/bioinformatics/btp616 19910308 PMC2796818

[B60] Barash Y Calarco JA Gao W Pan Q Wang X Shai O Blencowe BJ Frey BJ Deciphering the splicing code. Nature 2010 14 53 59 10.1038/nature09000 20445623

[B61] Yeo GW Coufal NG Liang TY Peng GE Fu X-D Gage FH An RNA code for the FOX2 splicing regulator revealed by mapping RNA-protein interactions in stem cells. Nat Struct Mol Biol 2009 14 130 137 10.1038/nsmb.1545 19136955 PMC2735254

[B62] Emilsson V Thorleifsson G Zhang B Leonardson AS Zink F Zhu J Carlson S Helgason A Bragi Walters G Gunnarsdottir S Mouy M Steinthorsdottir V Eiriksdottir GH Bjornsdottir G Reynisdottir I Gudbjartsson D Helgadottir A Jonasdottir A Jonasdottir A Styrkarsdottir U Gretarsdottir S Magnusson KP Stefansson H Fossdal R Kristjansson K Gislason HG Stefansson T Leifsson BG Thorsteinsdottir U Lamb JR Genetics of gene expression and its effect on disease. Nature 2008 14 423 428 10.1038/nature06758 18344981

[B63] Hsu Y-H Zillikens MC Wilson SG Farber CR Demissie S Soranzo N Bianchi EN Grundberg E Liang L Richards JB Estrada K Zhou Y van Nas A Moffatt MF Zhai G Hofman A van Meurs JB Pols HAP Price RI Nilsson O Pastinen T Cupples LA Lusis AJ Schadt EE Ferrari S Uitterlinden AG Rivadeneira F Spector TD Karasik D Kiel DP An integration of genome-wide association study and gene expression profiling to prioritize the discovery of novel susceptibility Loci for osteoporosis-related traits. PLoS Genet 2010 14 e1000977 10.1371/journal.pgen.1000977 20548944 PMC2883588

[B64] Hernandez DG Nalls MA Moore M Chong S Dillman A Trabzuni D Gibbs JR Ryten M Arepalli S Weale ME Zonderman AB Troncoso J O'Brien R Walker R Smith C Bandinelli S Traynor BJ Hardy J Singleton AB Cookson MR Integration of GWAS SNPs and tissue specific expression profiling reveal discrete eQTLs for human traits in blood and brain. Neurobiol Dis 2012 14 20 28 10.1016/j.nbd.2012.03.020 22433082 PMC3358430

[B65] Brooks AN Yang L Duff MO Hansen KD Park JW Dudoit S Brenner SE Graveley BR Conservation of an RNA regulatory map between Drosophila and mammals. Genome Res 2011 14 193 202 10.1101/gr.108662.110 20921232 PMC3032923

[B66] Shen S Park JW Huang J Dittmar KA Lu ZX Zhou Q Carstens RP Xing Y MATS: a Bayesian framework for flexible detection of differential alternative splicing from RNA-Seq data. Nucleic Acids Res 2012 14 e61 10.1093/nar/gkr1291 22266656 PMC3333886

[B67] Hansen KD Brenner SE Dudoit S Biases in Illumina transcriptome sequencing caused by random hexamer priming. Nucleic Acids Res 2010 14 e131 10.1093/nar/gkq224 20395217 PMC2896536

[B68] Li J Jiang H Wong WH Modeling non-uniformity in short-read rates in RNA-Seq data. Genome Biology 2010 14 R50 10.1186/gb-2010-11-5-r50 20459815 PMC2898062

[B69] Roberts A Trapnell C Donaghey J Rinn JL Pachter L Improving RNA-Seq expression estimates by correcting for fragment bias. Genome Biology 2011 14 R22 10.1186/gb-2011-12-3-r22 21410973 PMC3129672

[B70] Schwartz S Oren R Ast G Detection and removal of biases in the analysis of next-generation sequencing reads. PLoS ONE 2011 14 e16685 10.1371/journal.pone.0016685 21304912 PMC3031631

[B71] Warzecha CC Jiang P Amirikian K Dittmar KA Lu H Shen S Guo W Xing Y Carstens RP An ESRP-regulated splicing programme is abrogated during the epithelial-mesenchymal transition. EMBO J 2010 14 3286 3300 10.1038/emboj.2010.195 20711167 PMC2957203

[B72] Stephens M Balding DJ Bayesian statistical methods for genetic association studies. Nat Rev Genet 2009 14 681 690 19763151 10.1038/nrg2615

[B73] Veyrieras J-B Kudaravalli S Kim SY Dermitzakis ET Gilad Y Stephens M Pritchard JK High-resolution mapping of expression-QTLs yields insight into human gene regulation. PLoS Genet 2008 14 e1000214 10.1371/journal.pgen.1000214 18846210 PMC2556086

[B74] Gaffney D Veyrieras J-B Degner J Roger P-R Pai A Crawford G Stephens M Gilad Y Pritchard J Dissecting the regulatory architecture of gene expression QTLs. Genome Biol 2012 14 R7 10.1186/gb-2012-13-1-r7 22293038 PMC3334587

[B75] GLiMMPS. http://www.mimg.ucla.edu/faculty/xing/glimmps

[B76] Browning BL Browning SR A unified approach to genotype imputation and haplotype-phase inference for large data sets of trios and unrelated individuals. Am J Hum Genet 2009 14 210 223 10.1016/j.ajhg.2009.01.005 19200528 PMC2668004

[B77] R Core Development Team R: A Language and Environment for Statistical Computing. Vienna, Austria: R Foundation for Statistical Computing 2010

[B78] Suhre K Shin SY Petersen AK Mohney RP Meredith D Wagele B Altmaier E Deloukas P Erdmann J Grundberg E Hammond CJ de Angelis MH Kastenmuller G Kottgen A Kronenberg F Mangino M Meisinger C Meitinger T Mewes HW Milburn MV Prehn C Raffler J Ried JS Romisch-Margl W Samani NJ Small KS Wichmann HE Zhai G Illig T Spector TD Human metabolic individuality in biomedical and pharmaceutical research. Nature 2011 14 54 60 10.1038/nature10354 21886157 PMC3832838

[B79] Chambers JC Zhang W Sehmi J Li X Wass MN Van der Harst P Holm H Sanna S Kavousi M Baumeister SE Coin LJ Deng G Gieger C Heard-Costa NL Hottenga JJ Kuhnel B Kumar V Lagou V Liang L Luan J Vidal PM Mateo Leach I O'Reilly PF Peden JF Rahmioglu N Soininen P Speliotes EK Yuan X Thorleifsson G Alizadeh BZ Genome-wide association study identifies loci influencing concentrations of liver enzymes in plasma. Nat Genet 2011 14 1131 1138 10.1038/ng.970 22001757 PMC3482372

[B80] Evans DM Spencer CC Pointon JJ Su Z Harvey D Kochan G Oppermann U Dilthey A Pirinen M Stone MA Appleton L Moutsianas L Leslie S Wordsworth T Kenna TJ Karaderi T Thomas GP Ward MM Weisman MH Farrar C Bradbury LA Danoy P Inman RD Maksymowych W Gladman D Rahman P Morgan A Marzo-Ortega H Bowness P Gaffney K Interaction between ERAP1 and HLA-B27 in ankylosing spondylitis implicates peptide handling in the mechanism for HLA-B27 in disease susceptibility. Nat Genet 2011 14 761 767 10.1038/ng.873 21743469 PMC3640413

[B81] Scott LJ Muglia P Kong XQ Guan W Flickinger M Upmanyu R Tozzi F Li JZ Burmeister M Absher D Thompson RC Francks C Meng F Antoniades A Southwick AM Schatzberg AF Bunney WE Barchas JD Jones EG Day R Matthews K McGuffin P Strauss JS Kennedy JL Middleton L Roses AD Watson SJ Vincent JB Myers RM Farmer AE Genome-wide association and meta-analysis of bipolar disorder in individuals of European ancestry. Proc Natl Acad Sci USA 2009 14 7501 7506 10.1073/pnas.0813386106 19416921 PMC2678639

[B82] Ramsuran V Kulkarni H He W Mlisana K Wright EJ Werner L Castiblanco J Dhanda R Le T Dolan MJ Guan W Weiss RA Clark RA Karim SS Ahuja SK Ndung'u T Duffy-null-associated low neutrophil counts influence HIV-1 susceptibility in high-risk South African black women. Clin Infect Dis 2011 14 1248 1256 10.1093/cid/cir119 21507922 PMC3115278

[B83] Amin N Byrne E Johnson J Chenevix-Trench G Walter S Nolte IM Vink JM Rawal R Mangino M Teumer A Keers JC Verwoert G Baumeister S Biffar R Petersmann A Dahmen N Doering A Isaacs A Broer L Wray NR Montgomery GW Levy D Psaty BM Gudnason V Chakravarti A Sulem P Gudbjartsson DF Kiemeney LA Thorsteinsdottir U Stefansson K Genome-wide association analysis of coffee drinking suggests association with CYP1A1/CYP1A2 and NRCAM. Mol Psychiatry 2012 14 1116 1129 10.1038/mp.2011.101 21876539 PMC3482684

[B84] Peden JF Hopewell JC Saleheen D Chambers JC Hager J Soranzo N Collins R Danesh J Elliott P Farrall M A genome-wide association study in Europeans and South Asians identifies five new loci for coronary artery disease. Nat Genet 2011 14 339 344 10.1038/ng.782 21378988

[B85] Ochi H Maekawa T Abe H Hayashida Y Nakano R Kubo M Tsunoda T Hayes CN Kumada H Nakamura Y Chayama K ITPA polymorphism affects ribavirin-induced anemia and outcomes of therapy--a genome-wide study of Japanese HCV virus patients. Gastroenterology 2010 14 1190 1197 10.1053/j.gastro.2010.06.071 20637204

