# Supplementary material for: GLiMMPS: robust statistical model for regulatory variation of alternative splicing using RNA-seq data
Source: Genome Biol. 2013 Jul 22;14(7):R74. doi: 10.1186/gb-2013-14-7-r74 (PMC4054007; doi:10.1186/gb-2013-14-7-r74)

Supplementary Methods

Statistical models for sQTL analysis

Here we describe the different models used for sQTL analysis using RNA-Seq data.

1. Linear model (lm)

All previous sQTL analyses took this approach using either the absolute exon read counts [1] or the percentage of the exon reads among all reads in the gene [2]. In our analysis, we used the point estimate of exon inclusion level ($\psi = y/n$) from splice junction reads (Supplementary Figure S1). For individual *i*, g_{ij} denotes the genotype at SNP_j , then the model is:

$$y_i / n_i = \beta_0 + \beta_j g_{ij} + \varepsilon_i$$

2. Generalized linear model (glm)

Using exon reads directly would be confounded by variation in gene expression levels, making it difficult to distinguish real exon-level splicing QTLs from expression QTLs. Using point estimate of inclusion level (ψ) eliminates the information about the confidence level of the estimate reflected by the read counts that support it.

A natural solution is to use the reads information from both the exon inclusion and skipping isoforms instead of only a point estimate of the exon inclusion level in sQTL analysis. Given the observed splice junction read counts as in Supplementary Figure S1, we can assume that the splice junction reads supporting 2 different isoforms follow a binomial distribution. This leads to a hierarchical model:

$$y_i \mid \psi_i \sim Binomial(n_i, \psi_i),$$

$$\psi_i = \text{logit}^{-1}(\beta_0 + \beta_j g_{ij})$$

Where $\text{logit}^{-1}(\beta_0)$ is the baseline exon inclusion level, and β_j is the additive effect of SNP *j*. In generalized linear model, this would be to test the null hypothesis H_0: $\beta_j = 0$. We used glm function with logit link function in R package for this model.

3. Dispersion estimate

The simple logistic regression in glm assumes that ψ is correctly modeled and thus: $E(y_i) = n_i\psi_i$, and $Var(y_i) = n_i\psi_i(1-\psi_i)$. In real data, we would always have variation of the exon inclusion level across individuals in the same genotype group. Thus the observed variance would be larger than the expected variance which leads to overdispersion in the model. We can estimate the dispersion parameter ϕ, where $Var(y_i) = \phi n_i\psi_i(1-\psi_i)$. The estimate of ϕ can be obtained from the Pearson statistic [3]:

$$\hat{\phi} = \frac{\sum_i (y_i - n_i\hat{\psi}_i)^2 / (n_i\hat{\psi}_i(1-\hat{\psi}_i))}{m - p}$$

where m is the total number of individuals and p is the number of estimated parameters in the model. When there is no dispersion, ϕ should be equal to 1. To test the overdispersion in the CEU data set, for the top sQTLs (false positive rate < 1% based on permutations of individual labels) discovered from the glm model, we calculated ϕ and obtained the p-value from the χ^2 distribution with (m-p) degrees of freedom.

4. Generalized linear mixed model prediction of sQTL (GLiMMPS)

To deal with overdispersion, we modeled the extra variance of ψ as a random effect for each individual i in the model with random effects, $u_{ij} \sim N(0,\sigma_{uj}^2)$ [4]. Let $u_{ij} = \sigma_{uj} z_{ij}$, then $z_{ij} \sim N(0,1)$, β_j denoting the fixed effect for SNP j, which leads to a generalized linear mixed model:

$$\psi_i = \operatorname{logit}^{-1}(\beta_0 + \beta_j g_{ij} + \sigma_{uj} z_{ij}).$$

Thus the joint likelihood for β, σ_{uj} is given by:

$$L(\beta,\sigma_{uj}) = \prod_{i=1}^{m} \binom{n_i}{y_i} \int \frac{\exp\{(\beta_0 + \beta_j g_{ij} + \sigma_{uj} z_{ij})\}^{y_i}}{\left[1 + \exp\{(\beta_0 + \beta_j g_{ij} + \sigma_{uj} z_{ij})\}\right]^{n_i}} N(z_{ij}) dz_{ij},$$

where $N(\cdot)$ is the standard normal density. Laplace approximation was used for the parameter estimations and a likelihood ratio test was used to obtain the P-values for the fixed effect β_j for each SNP j.

Simulations

To compare the power and false positive rate of the various methods we discussed, we simulated the splice junction reads by mimicking the characteristics of the experimental data where possible. For each simulation, we simulated a sample of m=50 individuals which is close to the sample size of the experimental data. We simulated 10,000 replicates for each combination of parameters. To examine the effect of sequencing depth, we simulated the total number of splice junction reads (n) with mean value ($\bar{n}$) equals to 5, 10, 20, 50, and 80, respectively.

Because the total splice junction reads for each exon vary across individuals and they do not follow simple Poisson distribution, we used a Gamma-Poisson hierarchical model. First we generated the mean of Poisson distribution for each $\bar{n}$ value: $\lambda_i \sim Gamma(\alpha,\beta)$, where the shape parameter $\alpha = \bar{n}\beta$ and the rate parameter $\beta = 0.1$, which is the mean from the model fitting on experimental data. Then we generated n_i for individual i using Poisson distribution $n_i \sim Poisson(\lambda_i)$. After that, we simulated the genotypes (g_{ij}) for a random SNP j with the minor allele frequency (MAF) modeled by an exponential decay distribution with minimum MAF = 0.05 to obtain 3 genotypes AA, AB, BB. Then we simulated the baseline exon inclusion level ($\overline{\psi}_0$) for genotype AA according to a distribution Beta(0.35,0.81) that fits the observed pattern in the top sQTLs (false positive rate < 1% based on permutations of individual labels) identified by GLIMMPS from the

CEU data set (Supplementary Figure S4a). We simulated the difference of mean exon inclusion level of BB from AA ($\Delta\psi$) based on the mean and variance from the observed top sQTLs in the CEU data set (Supplementary Figure S4b). Assuming an additive model, we can obtain the mean ψ for genotype AB as well. Then for each genotype group, a noise was added according to normal distribution with the standard deviation sampled from the distribution observed in the top sQTLs in the CEU data set (Supplementary Figure S4c). After obtaining ψ_i for each individual, the inclusion isoform splice junction read counts were generated as: $y_i \sim Binomial(n_i, \psi_i)$.

Note that these simulations assume a normal distribution of ψ in each genotype group with the mean, genotype effect and noise mimicking the distributions from top sQTLs in the CEU data set. This is different from the logistic regression link function applied in GLiMMPS, which avoids biasing the simulation results in favor of our model.

Using simulation data with equal mean inclusion levels for three genotype groups and with various read depth, we evaluated the false positive rates (Type I errors) at 5% significance level. We also computed the receiver operating characteristic (ROC) curve by combining all the simulated data with null effects and various SNP effects.

sQTL analysis and estimation of FDR

For both CEU and CEU2 data sets, using each of the statistical models (lm, glm and GLiMMPS) described above, we carried out the analysis for each AS event using SNPs within 200kb of the target exon. To estimate the false discovery rate, we used the same approach as in [2]. For each exon, we permuted the individual label 10 times, recalculated the P-values, and recorded the minimum P-value for each exon for each permutation. This set of minimum P-values serves as the empirical null distribution for the P-values (denoted as p_0). We then compared the true distribution of minimum P-values (denoted as p_1) to this null distribution to obtain the estimate of FDR. For example, for FDR =0.1, we found a P-value cutoff z such that $P(p_0 < z)/P(p_1 < z) = 0.1$, where $P(p_0 < z)$ is the fraction of minimum P-values from permutation less than z and $P(p_1 < z)$ is the fraction of minimum P-values from observed data less than z. Partitioning the 10 permutations into 5 different sets yielded similar estimates of the FDR curve, suggesting our permutation procedure is sufficient to capture the null distribution genome-wide (Supplementary Figure S5).

References

1. Montgomery S, Sammeth M, Gutierrez-Arcelus M, Lach R, Ingle C, Nisbett J, Guigo R, Dermitzakis E: **Transcriptome genetics using second generation sequencing in a Caucasian population.** *Nature* 2010, **464:**773-777.
2. Pickrell J, Marioni J, Pai A, Degner J, Engelhardt B, Nkadori E, Veyrieras J-B, Stephens M, Gilad Y, Pritchard J: **Understanding mechanisms underlying human gene expression variation with RNA sequencing.** *Nature* 2010, **464:**768-772.
3. McCullagh P, Nelder J: *Generalized Linear Models.* Chapman and Hall; 1989.

4. Browne WJ, Subramanian SV, Jones K, Goldstein H: **Variance partitioning in multilevel logistic models that exhibit overdispersion.** *Journal of the Royal Statistical Society Series a-Statistics in Society* 2005, **168:**599-613.

Supplementary Figures

Supplementary Figure S1. Four types of alternative splicing events analyzed. Schematic diagrams of different types of alternative splicing events analyzed. The main target exon, mutually exclusively spliced exon, and neighboring constitutive exons are illustrated in black hatched box, grey hatched box, and open boxes. The exon inclusion and skipping splice junctions of the target alternatively spliced exon are depicted on the top and bottom as solid and dashed lines, respectively. Parameters used in the model are illustrated on the right, with y representing the effective read count from the exon inclusion isoform and n representing the total effective read count from both the exon inclusion and skipping isoforms.

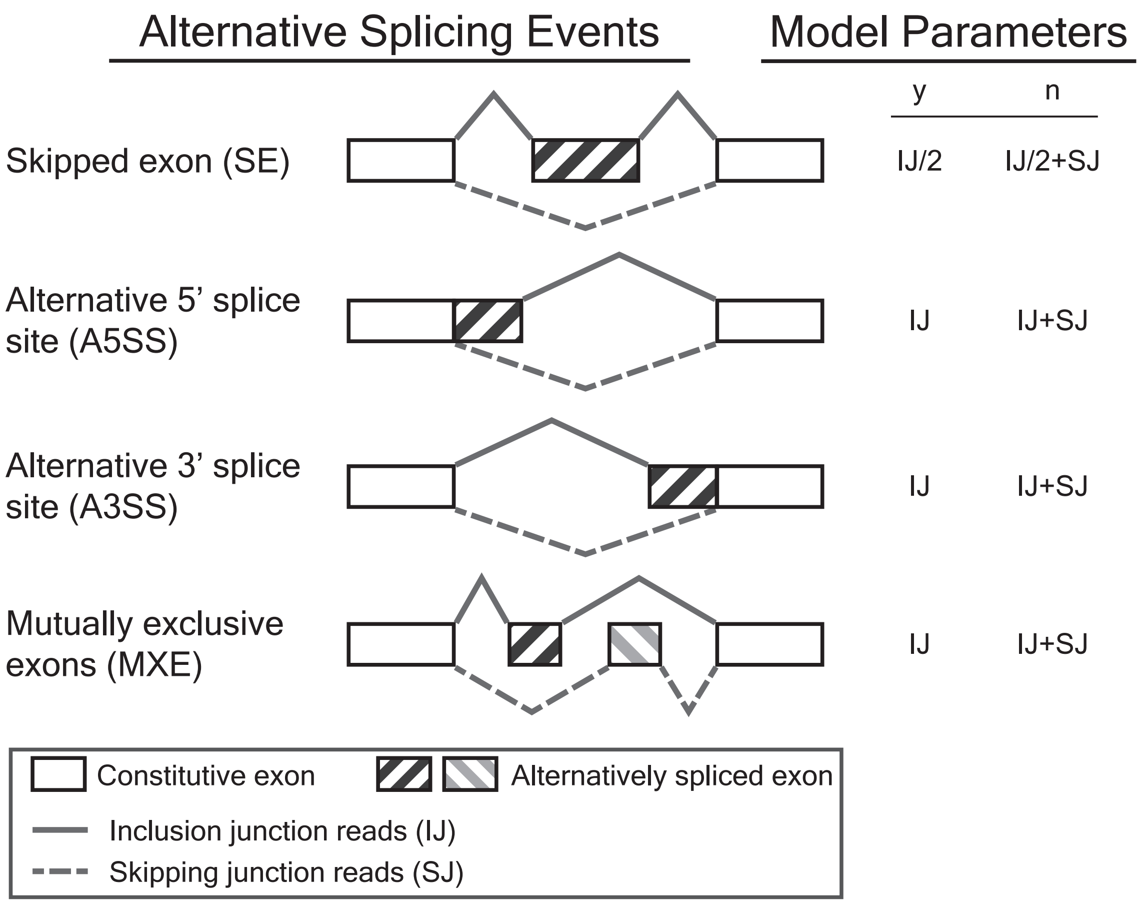

Supplementary Figure S2. Distribution of total splice junction read counts (n) across individuals in the CEU RNA-Seq data set. 20 randomly selected alternatively spliced exons from the CEU data set are shown in the plot.

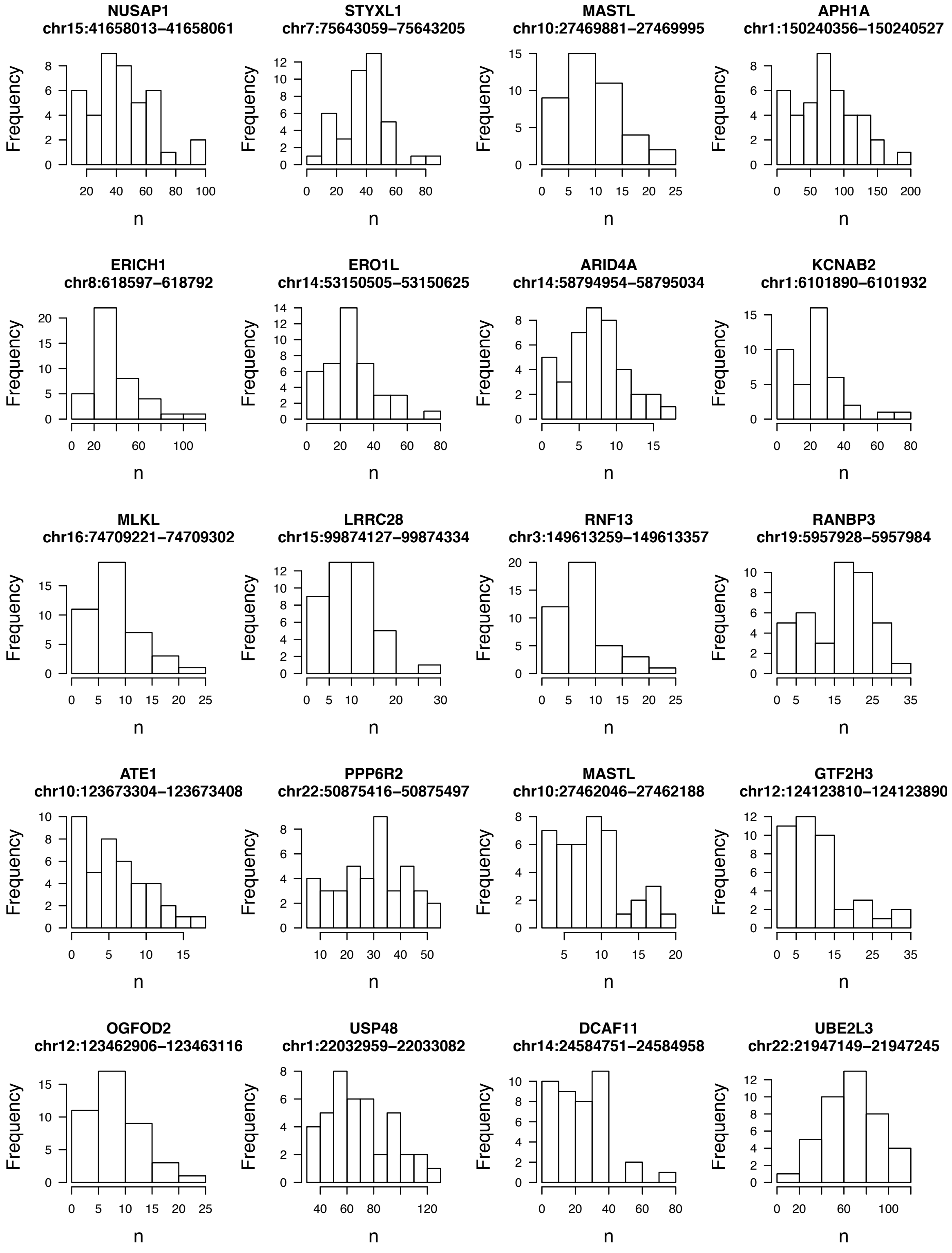

Supplementary Figure S3. Dispersion parameter estimate from top sQTLs (Type I error < 1%) from the glm model on the CEU data set. The estimate of the dispersion parameter ϕ for top sQTLs (false positive rate < 1%) from the glm model was obtained from the Pearson statistic, described in detail in Supplementary Methods. The distribution of the p-values for overdispersion of ϕ is shown on the right.

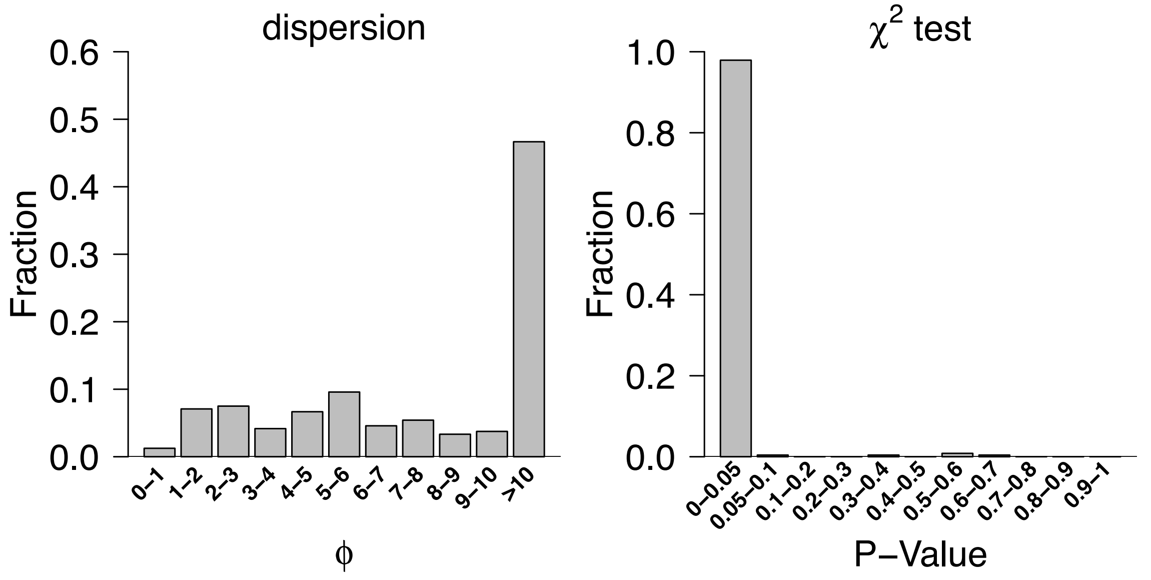

Supplementary Figure S4. Distributions of model parameters for top GLiMMPS sQTLs (false positive rate < 1%) in the CEU data set. (a) Distribution of the mean exon inclusion level ($\bar{\psi}_0$) in the baseline genotype group. The best Beta distribution fitted to the data is shown in the dashed line. **(b)** Distribution of the mean difference of exon inclusion levels ($\Delta\psi$) between the 2 homozygous genotypes. **(c)** Distribution of the standard deviation of exon inclusion level (Std (ψ)) of each genotype group.

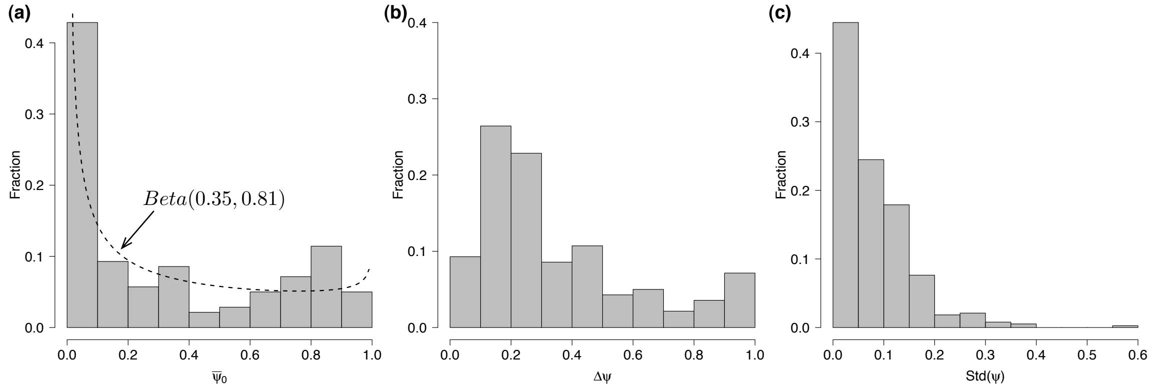

Supplementary Figure S5. FDR estimate based on the most significant P-value for each exon from the permuted data and observed data. The red line represents the FDR estimate from all 10 permutations. Five grey lines represent FDR estimates based on two permutations per line.

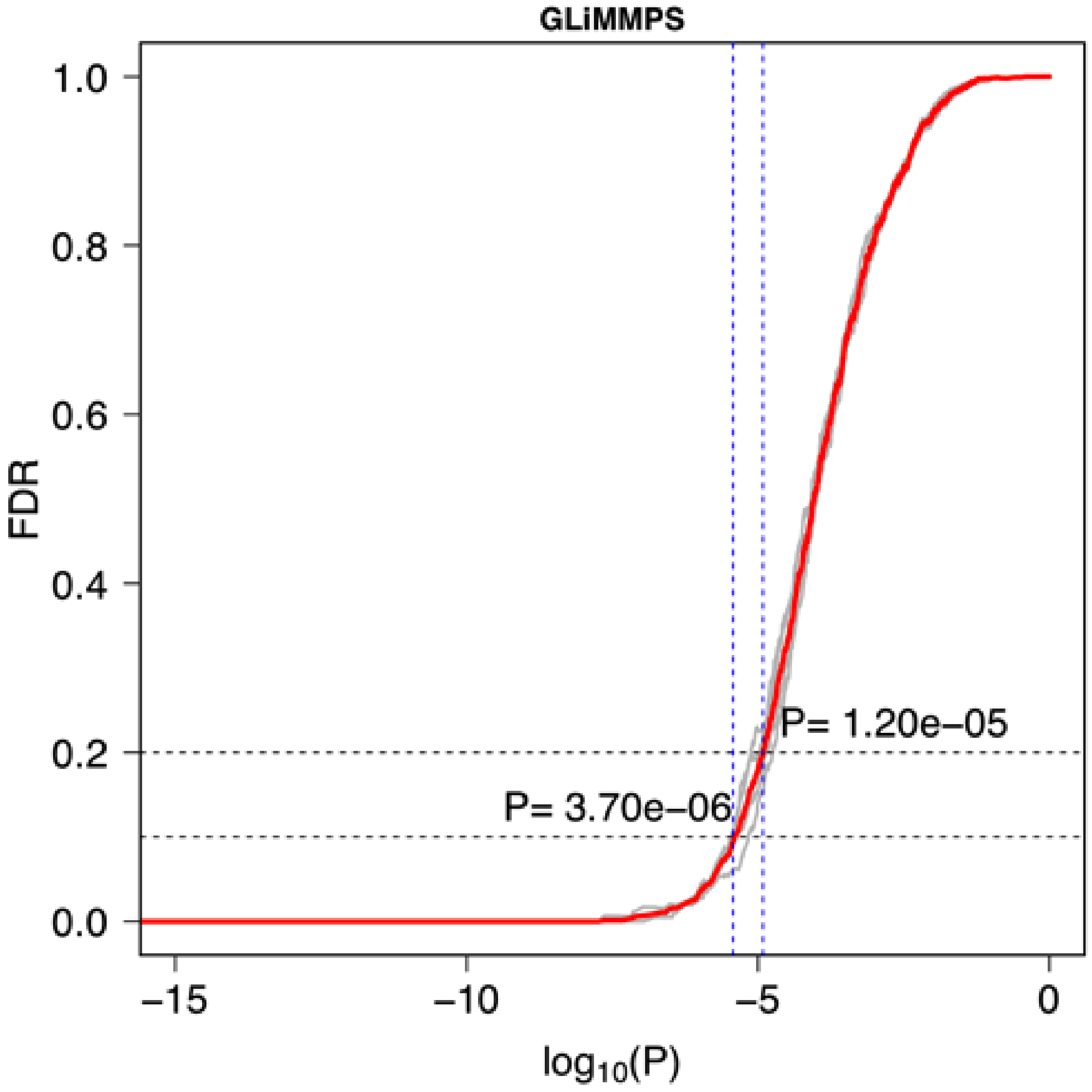

Supplementary Figure S6. Boxplots showing the correlation of exon inclusion levels (ψ) from RNA-Seq and RT-PCR with the most significant HapMap3 SNP. For improved illustration, individuals from the same genotype group are jittered on the X-axis, and semi-transparent box plots showing the median and quartiles are overlayed on top of the dot plot for each exon. In the RNA-Seq plot, the dot size for each individual is proportional to the total number of splice junction reads for the individual.

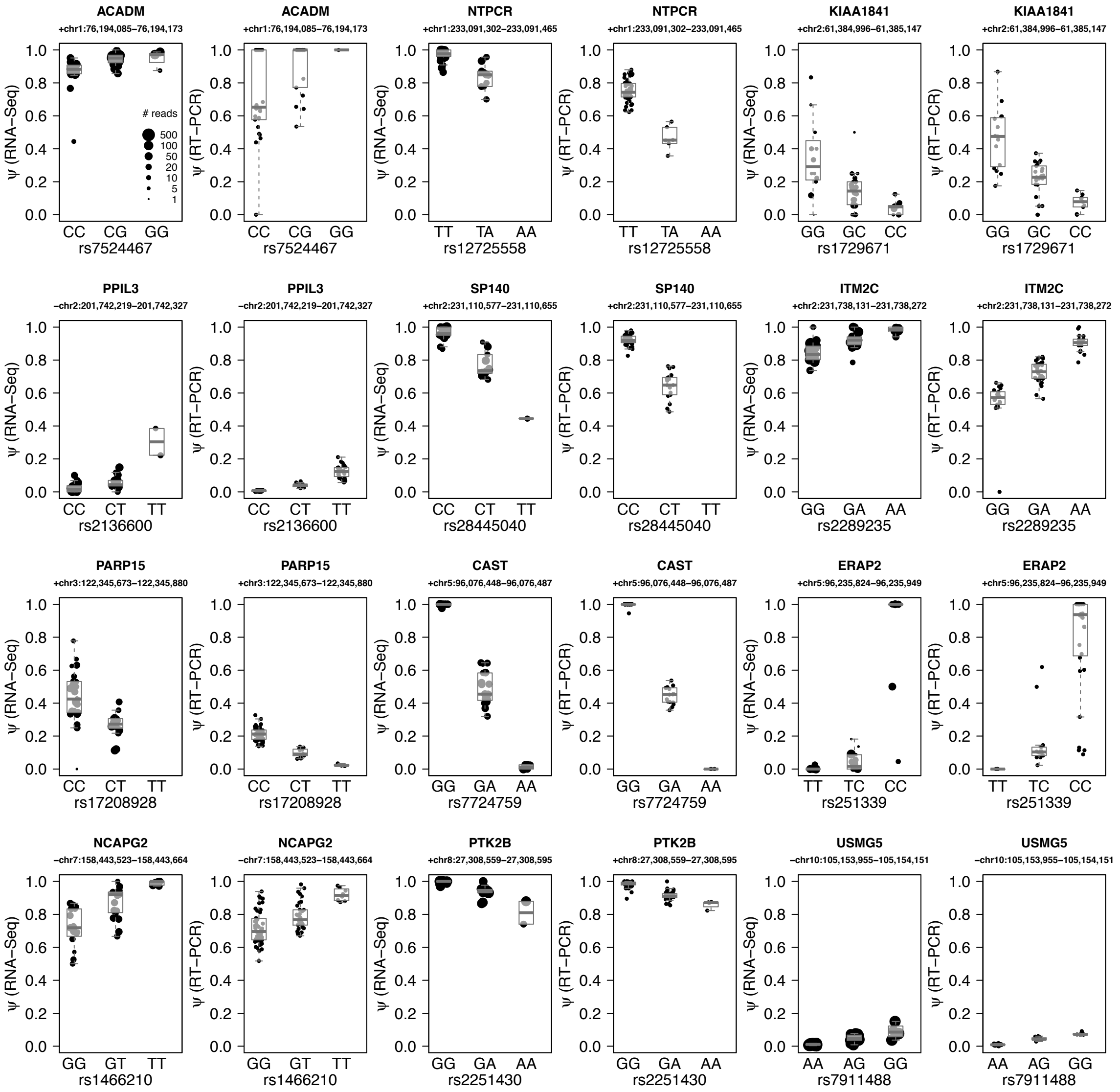

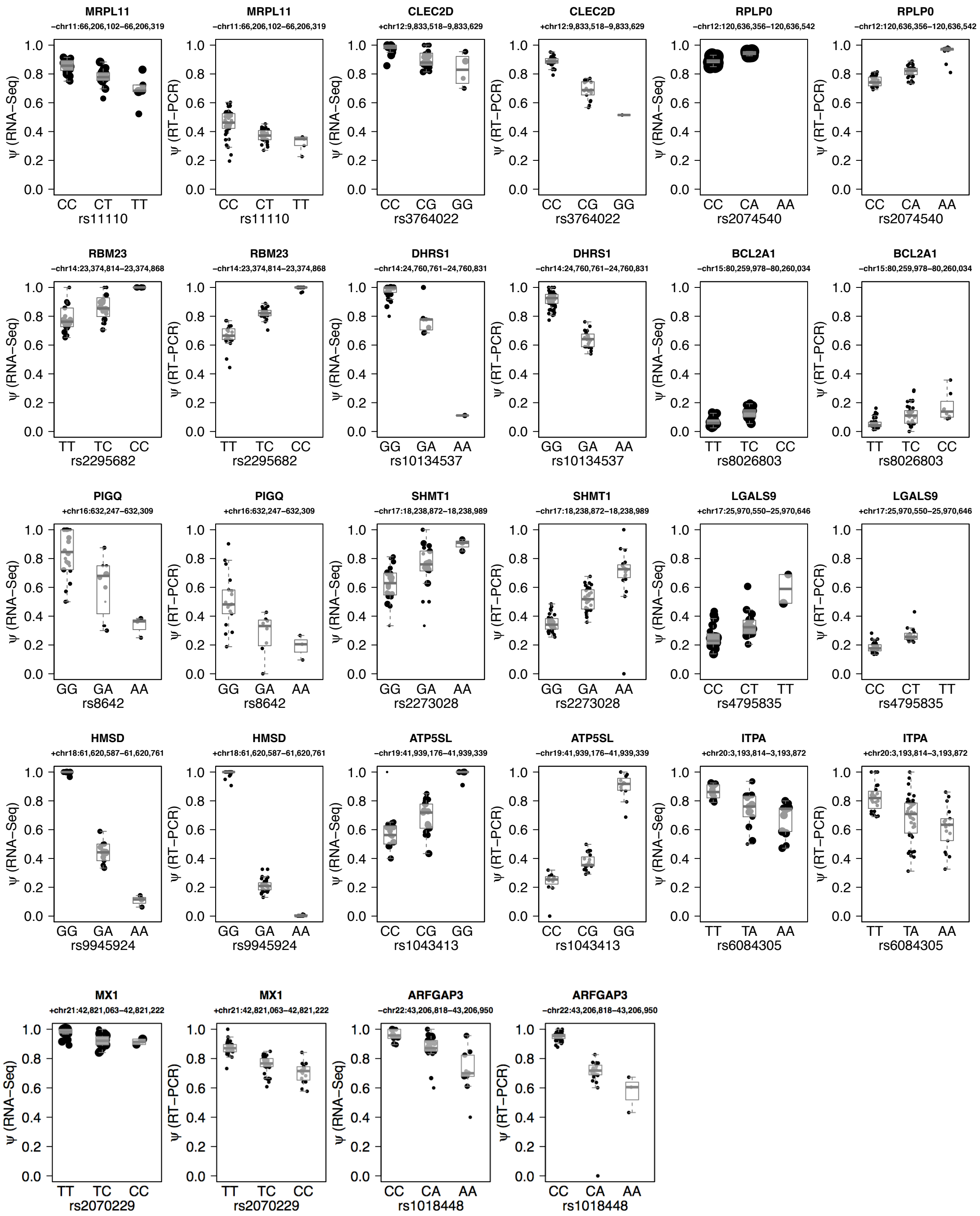
MRPL11
−chr11:66,206,102−66,206,319
ψ (RNA−Seq)
CC CT TT
rs11110
MRPL11
−chr11:66,206,102−66,206,319
ψ (RT−PCR)
CC CT TT
rs11110
CLEC2D
+chr12:9,833,518−9,833,629
ψ (RNA−Seq)
CC CG GG
rs3764022
CLEC2D
+chr12:9,833,518−9,833,629
ψ (RT−PCR)
CC CG GG
rs3764022
RPLP0
−chr12:120,636,356−120,636,542
ψ (RNA−Seq)
CC CA AA
rs2074540
RPLP0
−chr12:120,636,356−120,636,542
ψ (RT−PCR)
CC CA AA
rs2074540
RBM23
−chr14:23,374,814−23,374,868
ψ (RNA−Seq)
TT TC CC
rs2295682
RBM23
−chr14:23,374,814−23,374,868
ψ (RT−PCR)
TT TC CC
rs2295682
DHRS1
−chr14:24,760,761−24,760,831
ψ (RNA−Seq)
GG GA AA
rs10134537
DHRS1
−chr14:24,760,761−24,760,831
ψ (RT−PCR)
GG GA AA
rs10134537
BCL2A1
−chr15:80,259,978−80,260,034
ψ (RNA−Seq)
TT TC CC
rs8026803
BCL2A1
−chr15:80,259,978−80,260,034
ψ (RT−PCR)
TT TC CC
rs8026803
PIGQ
+chr16:632,247−632,309
ψ (RNA−Seq)
GG GA AA
rs8642
PIGQ
+chr16:632,247−632,309
ψ (RT−PCR)
GG GA AA
rs8642
SHMT1
−chr17:18,238,872−18,238,989
ψ (RNA−Seq)
GG GA AA
rs2273028
SHMT1
−chr17:18,238,872−18,238,989
ψ (RT−PCR)
GG GA AA
rs2273028
LGALS9
+chr17:25,970,550−25,970,646
ψ (RNA−Seq)
CC CT TT
rs4795835
LGALS9
+chr17:25,970,550−25,970,646
ψ (RT−PCR)
CC CT TT
rs4795835
HMSD
+chr18:61,620,587−61,620,761
ψ (RNA−Seq)
GG GA AA
rs9945924
HMSD
+chr18:61,620,587−61,620,761
ψ (RT−PCR)
GG GA AA
rs9945924
ATP5SL
−chr19:41,939,176−41,939,339
ψ (RNA−Seq)
CC CG GG
rs1043413
ATP5SL
−chr19:41,939,176−41,939,339
ψ (RT−PCR)
CC CG GG
rs1043413
ITPA
+chr20:3,193,814−3,193,872
ψ (RNA−Seq)
TT TA AA
rs6084305
ITPA
+chr20:3,193,814−3,193,872
ψ (RT−PCR)
TT TA AA
rs6084305
MX1
+chr21:42,821,063−42,821,222
ψ (RNA−Seq)
TT TC CC
rs2070229
MX1
+chr21:42,821,063−42,821,222
ψ (RT−PCR)
TT TC CC
rs2070229
ARFGAP3
−chr22:43,206,818−43,206,950
ψ (RNA−Seq)
CC CA AA
rs1018448
ARFGAP3
−chr22:43,206,818−43,206,950
ψ (RT−PCR)
CC CA AA
rs1018448

Supplementary Figure S7. Minigene experiments for testing causal sQTL SNPs. For each sQTL target exon, the gene symbol and exon coordinate in hg19 (with start position 0-based and end position 1-based) are shown on top. For each tested SNP, the dbSNP ID, genotypes, SNP annotation type, and GLiMMPS P-value are listed. The estimated exon inclusion levels of different minigene constructs are indicated below each gel picture. Each column represents the minigene construct corresponding to a particular genotype as indicated above the gel picture. The exon inclusion level is calculated from fluorescently labeled RT-PCR data as the intensity of the exon inclusion bands(s) over the total intensity of all exon inclusion and skipping bands. The last 4 exons marked in red have no tested SNPs causing more than 10% change in minigene exon inclusion levels. Universal primer sequences were used to test the inclusion level of target exon with the exception of *ITPA*. The *ITPA* minigene construct contains two adjacent exons (target exon 2 and the downstream exon 3) of *ITPA*. Both exons are alternatively spliced and are similar in size. Based on PCR product size alone, we cannot distinguish the transcript with target exon (exon 2) from the transcript with the downstream exon (exon 3), so we used target exon specific primers for RT-PCR analysis.

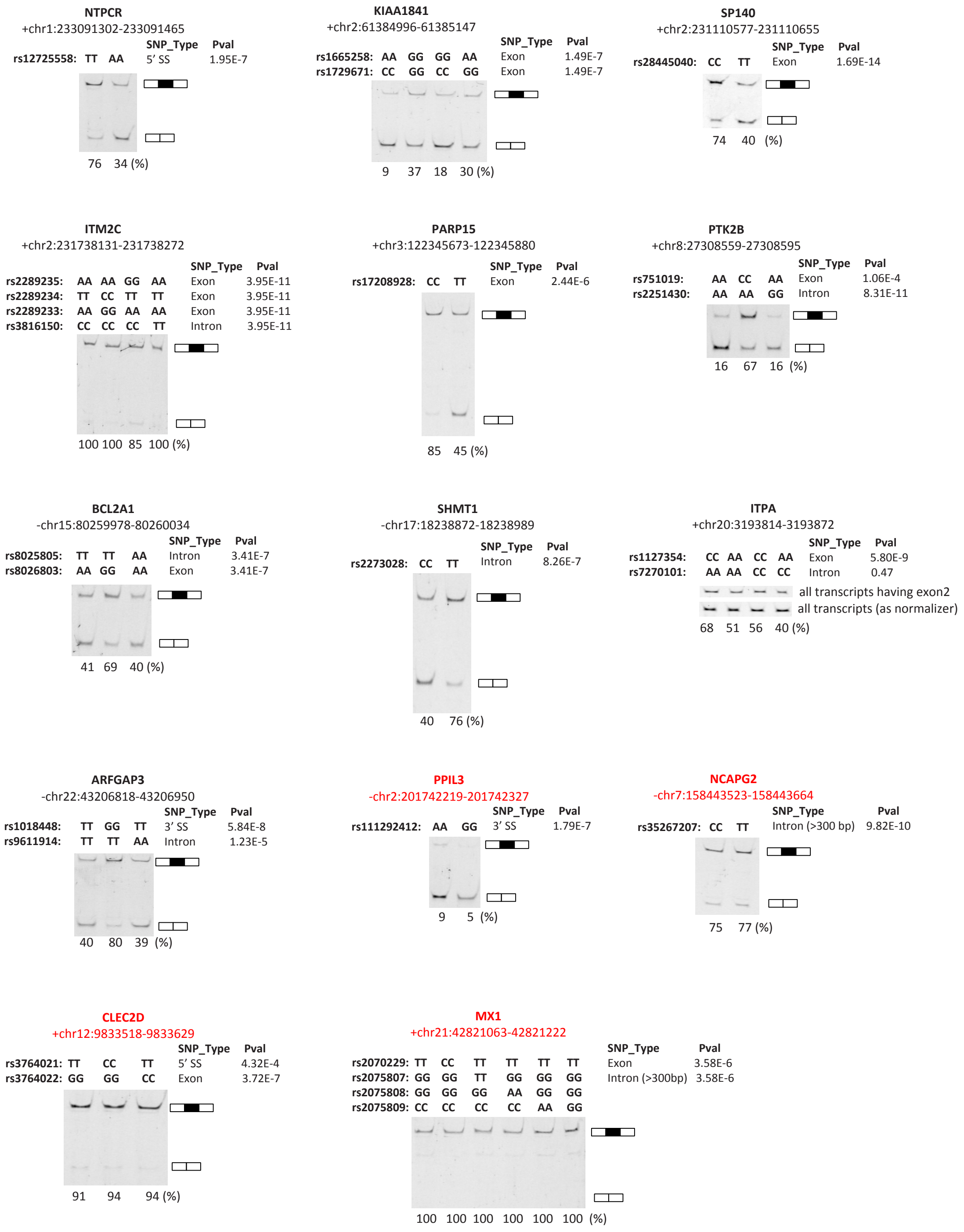
NTPCR
+chr1:233091302-233091465
SNP_Type Pval
rs12725558: TT AA 5' SS 1.95E-7
76 34 (%)
KIAA1841
+chr2:61384996-61385147
SNP_Type Pval
rs1665258: AA GG GG AA Exon 1.49E-7
rs1729671: CC GG CC GG Exon 1.49E-7
9 37 18 30 (%)
SP140
+chr2:231110577-231110655
SNP_Type Pval
rs28445040: CC TT Exon 1.69E-14
74 40 (%)
ITM2C
+chr2:231738131-231738272
SNP_Type Pval
rs2289235: AA AA GG AA Exon 3.95E-11
rs2289234: TT CC TT TT Exon 3.95E-11
rs2289233: AA GG AA AA Exon 3.95E-11
rs3816150: CC CC CC TT Intron 3.95E-11
100 100 85 100 (%)
PARP15
+chr3:122345673-122345880
SNP_Type Pval
rs17208928: CC TT Exon 2.44E-6
85 45 (%)
PTK2B
+chr8:27308559-27308595
SNP_Type Pval
rs751019: AA CC AA Exon 1.06E-4
rs2251430: AA AA GG Intron 8.31E-11
16 67 16 (%)
BCL2A1
-chr15:80259978-80260034
SNP_Type Pval
rs8025805: TT TT AA Intron 3.41E-7
rs8026803: AA GG AA Exon 3.41E-7
41 69 40 (%)
SHMT1
-chr17:18238872-18238989
SNP_Type Pval
rs2273028: CC TT Intron 8.26E-7
40 76 (%)
ITPA
+chr20:3193814-3193872
SNP_Type Pval
rs1127354: CC AA CC AA Exon 5.80E-9
rs7270101: AA AA CC CC Intron 0.47
all transcripts having exon2
all transcripts (as normalizer)
68 51 56 40 (%)
ARFGAP3
-chr22:43206818-43206950
SNP_Type Pval
rs1018448: TT GG TT 3' SS 5.84E-8
rs9611914: TT TT AA Intron 1.23E-5
40 80 39 (%)
PPIL3
-chr2:201742219-201742327
SNP_Type Pval
rs111292412: AA GG 3' SS 1.79E-7
9 5 (%)
NCAPG2
-chr7:158443523-158443664
SNP_Type Pval
rs35267207: CC TT Intron (>300 bp) 9.82E-10
75 77 (%)
CLEC2D
+chr12:9833518-9833629
SNP_Type Pval
rs3764021: TT CC TT 5' SS 4.32E-4
rs3764022: GG GG CC Exon 3.72E-7
91 94 94 (%)
MX1
+chr21:42821063-42821222
SNP_Type Pval
rs2070229: TT CC TT TT TT TT Exon 3.58E-6
rs2075807: GG GG TT GG GG GG Intron (>300bp) 3.58E-6
rs2075808: GG GG GG AA GG GG
rs2075809: CC CC CC CC AA GG
100 100 100 100 100 100 (%)

Supplementary Figure S8. sQTL signal overlapping with GWAS signal near gene *ATP5SL*. (a) The distribution of GLiMMPS P-values around the sQTL exon (exon 5) in gene *ATP5SL*. The black horizontal dashed line reflects the 10% FDR cutoff and red vertical lines mark the location of the sQTL exon. SNPs in linkage disequilibrium (r^2>0.8) with the GWAS SNP rs17318596 for height (blue asterisk) are shown in solid black dots, while other SNPs are shown in grey circles. The causal splicing SNP in exon 5 is shown in red triangle. Exon-intron structure is shown in the bottom with the GWAS SNP (rs17318596) and the causal splicing SNP (rs1043413) marked at corresponding locations. **(b)** Boxplot showing the significant association of rs1043413 with exon inclusion level (ψ) of the *ATP5SL* exon 5 estimated by the CEU RNA-Seq data set. The size of each dot is scaled by the total number of splice junction reads for that individual. **(c)** The same boxplot using exon inclusion level (ψ) measured by quantitative RT-PCR.

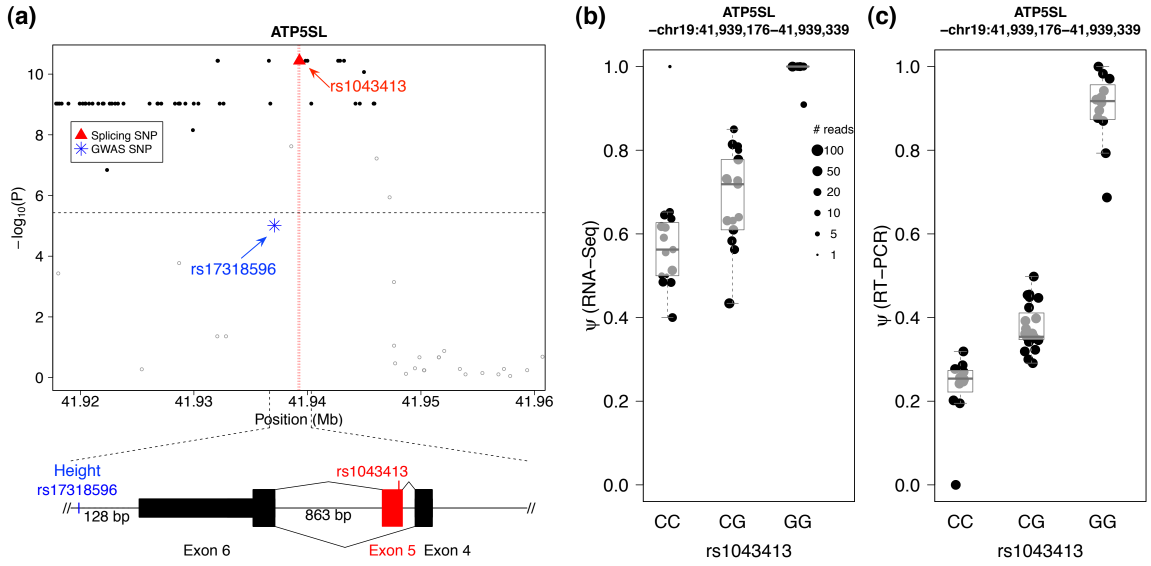

Supplementary Figure S9. sQTL signal overlapping with GWAS signal near gene *ITPA*. (a) The distribution of GLiMMPS P-values around the sQTL exon (exon 2) in gene *ITPA*. The black horizontal dashed line reflects the 10% FDR cutoff and red vertical lines mark the location of the sQTL exon. SNPs in linkage disequilibrium (r^2>0.8) with the GWAS SNP rs11697186 for response to hepatitis C treatment (blue asterisk) are shown in solid black dots, while other SNPs are shown in grey circles. The causal splicing SNP in exon 2 is shown in red triangle. Exon-intron structure is shown in the bottom with the GWAS SNP (rs11697186) and the causal splicing SNP (rs1127354) marked at corresponding locations. Because exon 2 and 3 are predominantly spliced together, both are colored in red. **(b)** Boxplot showing the significant association of rs1127354 with exon inclusion level (ψ) of the *ITPA* exon 2 estimated by the CEU RNA-Seq data set. The size of each dot is scaled by the total number of splice junction reads for that individual. **(c)** The same boxplot using exon inclusion level (ψ) measured by quantitative RT-PCR.

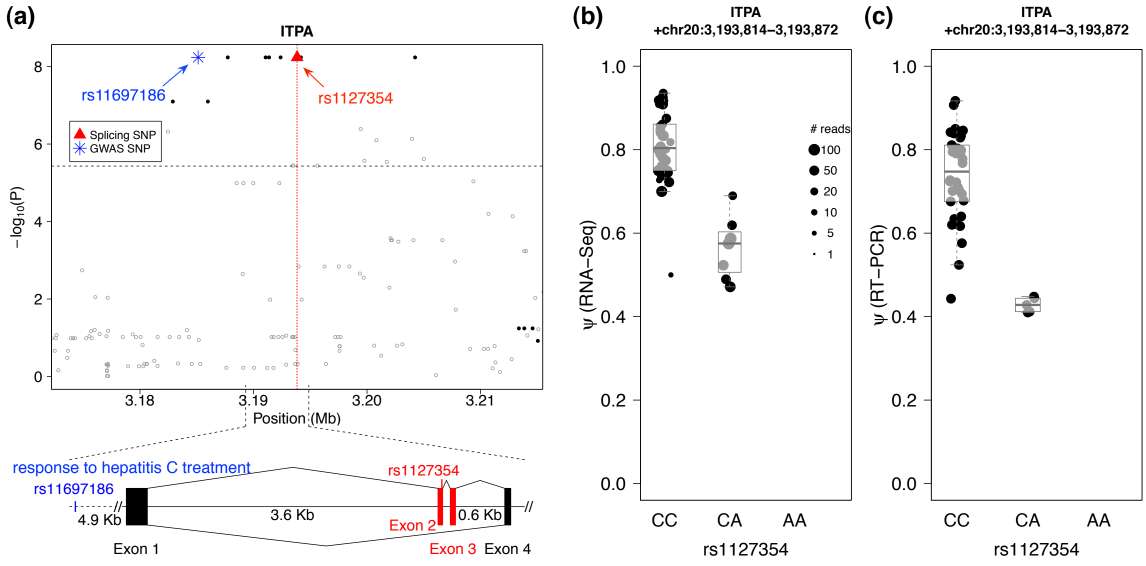

Supplement: Additional file 1 — Supplementary Methods and Supplementary Figures S1-S9. [file gb-2013-14-7-r74-S1.PDF]
